# Effect of Protein Kinase C delta (PKC-δ) Inhibition on the Transcriptome of Normal and Systemic Sclerosis Human Dermal Fibroblasts *In Vitro*

**DOI:** 10.1371/journal.pone.0027110

**Published:** 2011-11-11

**Authors:** Peter J. Wermuth, Sankar Addya, Sergio A. Jimenez

**Affiliations:** 1 Jefferson Institute of Molecular Medicine, Thomas Jefferson University, Philadelphia, Pennsylvania, United States of America; 2 Kimmel Cancer Center, Department of Cancer Biology, Jefferson Medical College, Thomas Jefferson University, Philadelphia, Pennsylvania, United States of America; University of Pittsburgh, United States of America

## Abstract

Previous studies demonstrated that protein kinase C- δ (PKC-δ) inhibition with the selective inhibitor, rottlerin, resulted in potent downregulation of type I collagen expression and production in normal human dermal fibroblasts and abrogated the exaggerated type I collagen production and expression in fibroblasts cultured from affected skin from patients with the fibrosing disorder systemic sclerosis (SSc). To elucidate the mechanisms involved in the ability of PKC-δ to regulate collagen production in fibroblasts, we examined the effects of PKC-δ inhibition on the transcriptome of normal and SSc human dermal fibroblasts. Normal and SSc human dermal fibroblasts were incubated with rottlerin (5 µM), and their gene expression was analyzed by microarrays. Pathway analysis and gene ontology analysis of differentially expressed genes in each comparison were performed. Identification of significantly overrepresented transcriptional regulatory elements (TREs) was performed using the Promoter Analysis and Interaction Network Toolset (PAINT) program. PKC-δ activity was also inhibited using RNA interference (siRNA) and by treating fibroblasts with a specific PKC-δ inhibitory cell permeable peptide. Differential gene expression of 20 genes was confirmed using real time PCR. PKC-δ inhibition caused a profound change in the transcriptome of normal and SSc human dermal fibroblasts *in vitro*. Pathway and gene ontology analysis identified multiple cellular and organismal pathways affected by PKC-δ inhibition. Furthermore, both pathway and PAINT analyses indicated that the transcription factor NFκB played an important role in the transcriptome changes induced by PKC-δ inhibition. Multiple genes involved in the degradation of the extracellular matrix components were significantly reduced in SSc fibroblasts and their expression was increased by PKC-δ inhibition. These results indicate that isoform-specific inhibition of PKC-δ profibrotic effects may represent a novel therapeutic approach for SSc and other fibrotic diseases.

## Introduction

The family of protein kinase C (PKC) serine/threonine kinases can be divided into three subclasses based on their distinctive structural and functional characteristics. The three subclasses of PKC are the conventional PKCs (cPKC; α, βI, βII, and γ), the novel PKC isoforms (nPKC; δ, ε, η, and θ), and the atypical PKC isoforms (aPKCs; ζ and ι/λ in human/mouse) [1]–[7] The cPKCs are diacylglycerol (DAG) sensitive and Ca^2+^ responsive, whereas the nPKCs are DAG sensitive but Ca^2+^ unresponsive, and the aPKCs are insensitive to both DAG and Ca^2+^.

PKC-δ influences a wide variety of cellular functions, most prominently, cellular growth and proliferation [8]–[9]. PKC-δ also participates in the initiation, progression and maintenance of inflammatory processes inducing NFκB activation, increased levels of intracellular adhesion molecule 1 (ICAM1), increased neutrophil adhesion, as well as, stimulation of the expression of cellular inhibitor of apoptosis (cIAP) protein family members and of proinflammatory mediators [10]–[12].

Numerous studies have shown that PKC-δ modulates the expression of collagen genes and increased PKC-δ has been associated with the development of pathologic tissue fibrosis [13]–[16]. For example, increased levels of PKC-δ have been reported in dermal fibroblasts from affected skin cultured from individuals afflicted with the fibrosing disorder systemic sclerosis or scleroderma (SSc) [13]. Furthermore, PKC-δ activation is necessary to mediate the stimulatory effect of Connective Tissue Growth Factor (CTGF) in cooperation with insulin/insulin growth factor 1 (IGF1) on collagen synthesis in SSc fibroblasts [14]. PKC-δ has also been shown to interact with components of the TGF-β signaling pathway. Other studies have shown that TGF-β activates PKC-δ which in turn positively regulates Smad3 transcriptional activity resulting in increased transcription of COL1A2 and fibronectin [17], [18].

Rottlerin, a derivative from *Mallotus phillipinensis*, the medicinal monkey face tree of India, causes potent and highly selective inhibition of PKC-δ with an IC50 of 3–6 µM, an effect 5–10 fold more potent than for PKC-α or PKC-β and nearly 13 to 33 fold more potent than for PKC-ε, γ, or η [19]. We previously showed that treatment of normal and SSc fibroblasts with rottlerin caused a potent decrease in the synthesis and production of type I and type III collagens in these fibroblasts [13]. In order to further analyze the mechanisms responsible, we examined the effects of PKC-δ inhibition with rottlerin on the transcriptome of normal and SSc-derived human dermal fibroblasts. These studies identified the transcription factor NFκB as a crucial participant in one of the most affected gene networks modulated by PKC-δ, an observation confirmed by an analysis of transcription factor binding sites demonstrating that the binding site for NFκB occurs at a significantly greater frequency in the differentially regulated genes than would be predicted by chance. We also found numerous genes that are involved in the regulation of synthesis or in the degradation of collagen and other extracellular matrix components to be differentially expressed in normal and SSc fibroblasts and modulated in response to PKC-δ inhibition, confirming the previous suggestions that PKC-δ plays an important role in the pathogenesis of tissue fibrosis in SSc and other fibrosing disorders [13].

## Materials and Methods

### Fibroblast cultures

Normal and SSc human dermal fibroblast cell lines were obtained from the Scleroderma Center Tissue Bank, Thomas Jefferson University. The SSc cell lines studied had been established from full-thickness skin biopsies obtained from 3 patients with SSc of recent onset (<18 months from the first appearance of clinically detectable skin induration). Cell lines obtained from 3 normal subjects were used as normal controls. The skin biopsy specimens from the SSc patients were obtained from the leading edge of the lesion on the forearms. The Institutional Review Board of Thomas Jefferson University approved the use of the tissues remaining after the diagnostic histopathologic studies for *in vitro* analyses. Since only discarded tissue was used, the IRB declared this study to be exempt and therefore neither written nor oral consent was required.

All SSc patients satisfied the criteria for classification of SSc and had the diffuse cutaneous clinical subset of the disease as defined by LeRoy *et al* [20]. Fibroblasts were expanded as described previously [13] from the frozen stocks and were used at passages 4–10.

### Treatment of cultured dermal fibroblasts with rottlerin

Normal and SSc human dermal fibroblasts were cultured in DMEM containing 10% FBS (Life Technologies Inc., Grand Island, New York, USA), 1% vitamins, 2 mM glutamine, antibiotics, and fungizone in 100 mm plates until confluent, then preincubated for 24 h with 40 µg/ml ascorbic acid phosphate magnesium salt n-hydrate (Sigma-Aldrich, St. Louis, MO) to optimize collagen production. Fibroblasts were then incubated for 24 h in fresh media supplemented with 40 µg/ml ascorbic acid and a final concentration of 5 µM rottlerin (Biomol Research Laboratories Inc., Plymouth Meeting, PA), a concentration previously demonstrated to induce maximal effects in human dermal fibroblasts without causing cellular toxicity [13].

### RNA Interference

Normal and SSc human dermal fibroblasts were cultured in 6 well plates until confluent. Transfection of 10 nM short interfering RNA (siRNA) directed against PKC-δ was performed using the HiPerFect reagent (Qiagen, Valencia CA) according to the manufacturer's protocol. At 24 h after siRNA transfection, fibroblasts were incubated for 24 h with 40 µg/ml ascorbic acid phosphate magnesium salt n-hydrate (Sigma-Aldrich, St. Louis, MO) to optimize collagen production. The siRNA target sequences were: PKC-δ - AACTCTACCGTGCCACGTTT; Control – CCGGGACACTATATTCCAGAA.

### Treatment of cultured dermal fibroblasts with a PKC-δ cell permeable peptide inhibitor

Normal and SSc human dermal fibroblasts were cultured as previously described in 6 well plates until confluent. Cultures were then incubated for 24 h with 10 µM of a control peptide or with 10 µM of a cell permeable inhibitory peptide specific for PKC-δ (Mimotopes Pty Ltd, Clayton Victoria, AU). Fresh media were added and fibroblasts were then incubated for 24 h with a second 10 µM aliquot of peptide and with 40 µg/ml ascorbic acid phosphate magnesium salt n-hydrate (Sigma-Aldrich, St. Louis, MO) to optimize collagen production. The control peptide consists of a dimer of the TAT protein transduction domain (PTD) [21] and its sequence was: TAT Control – RRRQRRKKRGYCCYGRKKRRQRRR. The PKC-δ peptide sequence is the same described in Chen et al., and consists of the N-terminal portion of the C2 domain (aa2-10) of PKC-δ fused to the TAT peptide [22] and its sequence was: PKD-TAT – RRRQRRKKRGYCCSFNSYELGSL.

### Microarray analysis

Following exposure to rottlerin, DNA-free total RNA was isolated utilizing the RNeasy micro kit (Qiagen, Valencia, CA), according to the manufacturer's protocol. DNase-treated total RNA was ethanol precipitated and quantified on a NanoDrop ND-1000 spectrophotometer and RNA quality was analyzed on an Agilent 2100 bioanalyser (Agilent Technologies, Palo Alto, CA). Double-stranded cDNA was synthesized using T7 Oligo dT (Integrated DNA Technologies, Coralville, IA) and Superscript II double-stranded cDNA Kit (Invitrogen, Carlsbad, CA). Biotinylated cRNA was prepared using the BioArray High Yield RNA Transcript Labeling Kit (Enzo Diagnostics, Farmingdale, NY). The labeled cRNA was fragmented by heat and ion-mediated hydrolysis and was hybridized to the Human Genome HU133A oligonucleotide array GeneChip (Affymetrix) which contains ∼500,000 spots comprised of 22,283 different probe sets representing 14,397 unique genes. The arrays were washed and stained using a GeneChip Fluidic Station 450 (Affymetrix), and hybridization signals were amplified utilizing antibody amplification with goat IgG (Sigma-Aldrich, Saint Louis, MO) and anti-streptavidin biotinylated antibody (Vector Laboratories, Burlingame, CA) and then were scanned using an Affymetrix GeneChip Scanner 3000, using GeneChip Operating Software (GCOS) version 3.0. Background correction and normalization were done using a Robust Multichip Average (RMA) with Genespring V 10.0 software (Agilent Technologies, Santa Clara, CA). Volcano plots were used to identify differentially expressed genes using the parametric testing assuming equal variances (based on the results of a Student's two-sample t-test for two groups).

Four different comparisons were performed: normal untreated fibroblasts versus normal fibroblasts treated with rottlerin, SSc untreated fibroblasts versus rottlerin treated SSc fibroblasts, normal untreated fibroblasts versus SSc untreated fibroblasts, and normal fibroblasts treated with rottlerin versus rottlerin treated SSc fibroblasts. The list of differentially expressed genes for each comparison was loaded into Ingenuity Pathway Analysis (IPA) 5.0 software (www.ingenuity.com) to perform biological network and functional analyses.

### Transcriptional regulatory network analysis

Promoter analysis was performed using the Promoter Analysis and Interaction Network Toolset (PAINT) v3.9 which contains a database of promoter sequences (UpstreamDB) constructed for all known and putative annotated genes in the Ensembl genome database for *Homo sapiens*, version 49, cross referenced with Unigene build #213 [23], [24]. This encompasses 32,559 promoter sequences in the Ensembl database cross-referenced to 19,423 promoter sequences in the Unigene database. Promoter analysis for putative transcriptional regulatory elements (TREs) which serve as binding sites for known transcription factors was performed employing the MATCH algorithm which scores potential matches based on the degree of similarity of a putative binding site to known binding sites using known binding site sequences contained in the TRANSFAC public database version 6.0. This analysis allows for the identification of common upstream transcription factor binding sequences in a list of genes and compares the prevalence of sequences within the set to that expected to be found by chance in a given list of TREs. PAINT analysis is a function of upstream sequence information and is independent of gene expression levels.

Each group of genes from the microarray analysis categories was tested for significantly overrepresented TREs against the entire set of genes in the Human Genome U133 Plus 2.0 array, which represent the global set of TREs affected by the experimental conditions. The default settings of the parameters of the program were utilized in the analysis. Statistical significance for TRE overrepresentation was set at p<0.05 with additional filtering performed by setting the false discovery rate (FDR) at 0.3. Identification of statistically significant enrichment of a specific TRE within a particular expression cluster indicates a role for the cognate transcription factor in the coordinate regulation of genes in that cluster.

### Validation of microarray results utilizing RT-PCR

A selected subset of differentially expressed genes was chosen for independent verification by SYBR Green-based, real-time RT-PCR (Applied Biosystems, Foster City, CA) following a standard amplification protocol on an ABI Prism 7900 Sequence Detection System (Applied Biosystems) using β-actin as an internal reference standard. Primer pairs (Integrated DNA Technologies, Coralville, IA) for representative genes from the analyzed data are listed in **Table S1**. Relative quantification was assessed by arbitrarily setting the expression level of the saline negative control at 100 and by expressing changes in transcript levels of other samples relative to this control sample. Relative differences in each PCR sample were corrected using human β-actin mRNA as an endogenous control. Real-time PCR values reflect the mean and standard deviation of triplicate samples. The statistical significance of the real-time PCR data was assessed by Student's two-tailed t test. *P* values less than 0.05 were considered significant.

### Accession Numbers

The entire dataset discussed in this paper is compliant to the Minimum Information About a Microarray Experiment (MIAME) criteria and has been deposited at Gene Expression Omnibus (http://www.ncbi.nlm.nih.gov/geo/query/acc.cgi?acc=GSE23741) under accession number GSE23741 (platform ID: GPL570; dataset IDs: GSM585874-GSM585885).

## Results

### Global gene expression of cultured normal and SSc dermal fibroblasts induced by PKC-δ inhibition

**Figure 1:**
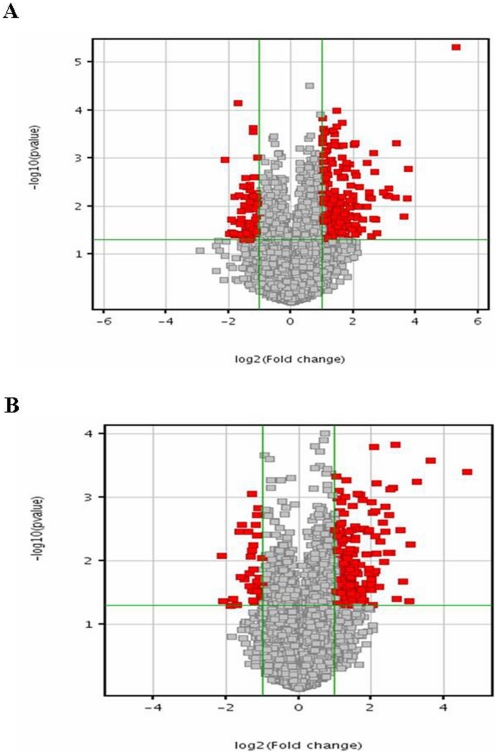
Volcano plot of differentially expressed transcripts. (**A**) Normal untreated vs normal rottlerin-treated fibroblasts. (**B**) SSc untreated vs SSc rottlerin-treated fibroblasts. A volcano plot of genes differentially expressed in human dermal fibroblasts exposed to rottlerin. The X-axis represents the log^2^ values of the fold change observed for each transcript whereas the Y axis depicts the log^10^ values of the p value of the significance tests between replicates for each transcript. Genes that demonstrate a 2 fold or greater difference in expression at a p value<0.05 in rottlerin treated cells compared to untreated cells are displayed in red. Dots representing genes of interest validated by RT-PCR are labeled.

**Figure 2:**
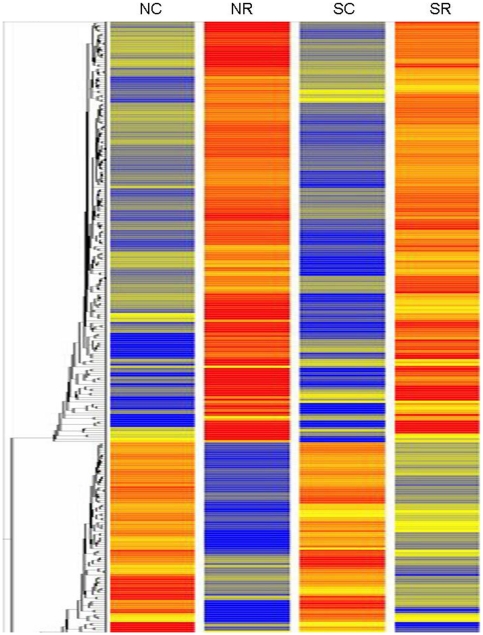
Heat map and dendrogram of differentially expressed transcripts. GeneSpring analysis of normal and SSc human dermal fibroblasts exposed to rottlerin. RNA was isolated from replicate samples of normal human dermal fibroblasts incubated under control conditions (NC) or cultured with 5 µM rottlerin (NR), or from SSc derived human dermal fibroblasts under control conditions (SC) or cultured with 5 µM rottlerin (SR), labeled and applied to Affymetrix human U133 2.0 Plus microarrays. Dendrograms are reflective of the genes with a differential expression of >2 fold in the two experimental conditions. In the dendrogram shown, a shorter arm indicates higher similarity, whereas a longer arm indicates lower similarity. Genes with significant expression (p<0.05) between untreated and rottlerin treated fibroblasts were hierarchically clustered by similarity in expression profile. The resulting heat map of the dendrogram tree reveals groups of genes with high (red) expression levels, low expression levels (blue) or background expression levels (yellow).

Transcriptome profiling was performed employing microarray analysis on total RNA isolated from untreated and rottlerin-exposed human dermal fibroblasts derived from normal skin biopsies or from biopsies of affected skin from patients with diffuse SSc. Following normalization of hybridization intensities using the RMA (Robust Multichip Array) algorithm in the Genespring 7.3.1 software, the average expression level for each gene was calculated from biological duplicates. The values were plotted in a Volcano plot analyzing expression patterns in untreated normal fibroblasts versus rottlerin treated normal fibroblasts in **Figure 1a** and in untreated SSc fibroblasts versus rottlerin treated SSc fibroblasts in **Figure 1b** using the –log_10_[p-value] and –log_2_[fold change]. These assessments allowed filtering of the differentially expressed genes at a particular p-value. In order to discover specific patterns hierarchical clustering (Pearson correlation) was applied to the expression profiles of the total group of differentially expressed transcripts (**Figure 2**). A summary of the number of differentially expressed transcripts is depicted in **Table 1**.

**Table 1:** Number of differentially expressed transcripts in control fibroblasts vs rottlerin exposed human dermal fibroblasts derived from normal or SSc patients at different statistical criteria using Volcano plot as a filter.

	Upregulated	Downregulated	Total
**Transcripts Differentially Regulated by Rottlerin, 2 Fold, p<0.05**			
Untreated vs Rottlerin	320	113	433
Common Rottlerin Responsive	128	19	242
Rottlerin Responsive in Normal Cells Only	117	74	191
Rottlerin Responsive in SSc Cells Only	75	20	95
**Transcripts Differentially Regulated in SSc Fibroblasts, 2 Fold, p<0.05**			
Normal vs SSc	34	41	75
Common in Untreated and Rottlerin-treated Cells	10	5	15
Differentially Expressed in Untreated	10	18	28
Differentially Expressed in Rottlerin-treated	14	18	32
**Transcripts Differentially Regulated in SSc Fibroblasts, 1.5 Fold, p<0.10**			
Normal vs SSc	191	169	360
Common in Untreated and Rottlerin-treated Cells	38	23	61
Differentially Expressed in Untreated	62	72	134
Differentially Expressed in Rottlerin-treated	91	74	165

Exposure of normal and SSc fibroblasts to rottlerin induced changes of 2 fold or greater in the expression level of a total of 433 gene transcripts at a significance level of p<0.05, of which 320 were upregulated and 113 were downregulated. The upregulation of nearly 3 times more genes than those that are downregulated indicate that PKC-δ functions primarily as a transcriptional repressor in human dermal fibroblasts. Interestingly, the response of the transcriptome to rottlerin exposure differed dramatically depending upon the origin of the dermal fibroblasts. Of the differentially regulated transcripts, the expression levels of a total of 147 (128 upregulated and 19 downregulated) were changed in both normal and SSc cultured dermal fibroblasts. However, a total of 192 transcripts (117 upregulated and 75 downregulated) were expressed differentially only in fibroblasts derived from normal donors whereas expression levels changed for a total of 95 transcripts (75 upregulated and 20 downregulated) only in fibroblasts derived from SSc donors. A partial list of the most upregulated transcripts is displayed in **Table 2** and a partial list of the most downregulated transcripts is displayed in **Table 3**.

**Table 2:** Selected upregulated rottlerin responsive transcripts.

Gene Symbol	Description	Fold Change
**Transcripts Upregulated by Rottlerin in Normal and SSc cells**		
GDF15	growth differentiation factor 15	24.9
TRIB3	tribbles homolog 3 (Drosophila)	12.4
DDIT4	DNA-damage-inducible transcript 4	9.6
ASNS	asparagine synthetase	8.4
SLC7A11	solute carrier family 7 member 11	8.1
SLC7A5	solute carrier family 7 member 5	7.3
TRIB3	tribbles homolog 3 (Drosophila)	7.0
SLC7A11	solute carrier family 7, member 11	6.5
PSAT1	phosphoserine aminotransferase 1	6.2
CTH	cystathionase (cystathionine gamma-lyase)	6.0
SLC22A15	solute carrier family 22, member 15	5.7
PSAT1	phosphoserine aminotransferase 1	5.7
**Transcripts Upregulated by Rottlerin in Normal Cells Only**		
IL8	Interleukin 8	6.0
ITGB3	integrin, beta 3	4.4
SLC38A1	solute carrier family 38, member 1	4.4
CBS	cystathionine-beta-synthase	4.3
RRAGD	Ras-related GTP binding D	4.2
CD55	CD55 molecule	4.2
AKR1C1	Aldo-keto reductase family 1, member C1	3.8
PION	pigeon homolog (Drosophila)	3.6
VEGFA	vascular endothelial growth factor A	3.4
SLC4A7	solute carrier family 4, member 7	3.4
BEX4	brain expressed, X-linked 4	3.3
**Transcripts Upregulated by Rottlerin in SSc Cells Only**		
THBD	Thrombomodulin	5.1
THBD	Thrombomodulin	5.0
SLC6A15	solute carrier family 6 member 15	4.4
THBD	Thrombomodulin	4.2
PTGS1	prostaglandin-endoperoxide synthase 1	3.6
IGDCC4	Ig superfamily, DCC subclass, member 4	3.3
JARID2	jumonji, AT rich interactive domain 2	3.1
GADD45A	growth arrest and DNA-damage-induc., 45α	2.9
PTGS2	prostaglandin-endoperoxide synthase 2	2.9
IL20RB	Interleukin 20 receptor beta	2.9
NAMPT	nicotinamide phosphoribosyltransferase	2.8

Fold change indicates the difference between untreated normal or SSc cells compared to rottlerin treated normal or SSc cells. The entire dataset discussed in this paper is deposited at Gene Expression Omnibus (http://www.ncbi.nlm.nih.gov/geo/query/acc.cgi?acc=GSE23741) under accession number GSE23741.

**Table 3:** Selected downregulated rottlerin responsive transcripts.

Gene Symbol	Description	Fold Change
**Transcripts Downregulated by Rottlerin in Normal and SSc cells**		
C5orf13		−4.4
SOX9	SRY (sex determining region Y)-box 9	−4.3
SOX9	SRY (sex determining region Y)-box 9	−3.8
GBP1	Guanylate binding protein 1, IFN-inducible	−3.0
GBP1	Guanylate binding protein 1, IFN-inducible	−2.9
C5orf13	chromosome 5 open reading frame 13	−2.6
CXCL12	chemokine (C-X-C motif) ligand 12	−2.5
CARHSP1	calcium regulated heat stable protein 1	−2.5
HUNK	hormonally up-reg. Neu-associated kinase	−2.4
METTL7A	methyltransferase like 7A	−2.4
TNFRSF19	TNF receptor superfamily, member 19	−2.4
HELLS	helicase, lymphoid-specific	−2.4
**Transcripts Downregulated by Rottlerin in Normal Cells Only**		
FAM65B	family w/sequence similarity 65, memb.B	−3.5
SHROOM3	shroom family member 3	−3.2
		−3.1
ST6GALNAC5	Alpha-2,6 sialyltransferase	−3.1
PSRC1	proline/serine-rich coiled-coil 1	−3.1
SOCS2	suppressor of cytokine signaling 2	−3.0
MYLIP	myosin reg. light chain interacting protein	−3.0
MRVI1	murine retrovirus integ. site 1 homolog	−2.8
GNG2	guanine nucleotide binding protein, γ 2	−2.8
MKI67	antigen identified by MAb Ki-67	−2.8
C5orf13	chromosome 5 open reading frame 13	−2.7
**Transcripts Downregulated by Rottlerin in SSc Cells Only**		
CXCL6	chemokine (C-X-C motif) ligand 6	−3.5
CLIC3	chloride intracellular channel 3	−3.3
PHACTR3	phosphatase and actin regulator 3	−3.1
DIRAS3	DIRAS family, GTP-binding RAS-like 3	−3.1
FN1	fibronectin 1	−2.6
MCM4	minichromosome maintenance complex 4	−2.6
TEAD2	TEA domain family member 2	−2.4
OLFML1	olfactomedin-like 1	−2.4
MCM5	minichromosome maintenance complex 5	−2.3
GINS4	GINS complex subunit 4 (Sld5 homolog)	−2.3
RAB7B	RAB7B, member RAS oncogene family	−2.2

Fold change indicates the difference between untreated normal or SSc cells compared to rottlerin treated normal or SSc cells. The entire dataset discussed in this paper is deposited at Gene Expression Omnibus (http://www.ncbi.nlm.nih.gov/geo/query/acc.cgi?acc=GSE23741) under accession number GSE23741.

### Biological network and functional analysis of transcripts differentially regulated by rottlerin

**Figure 3:**
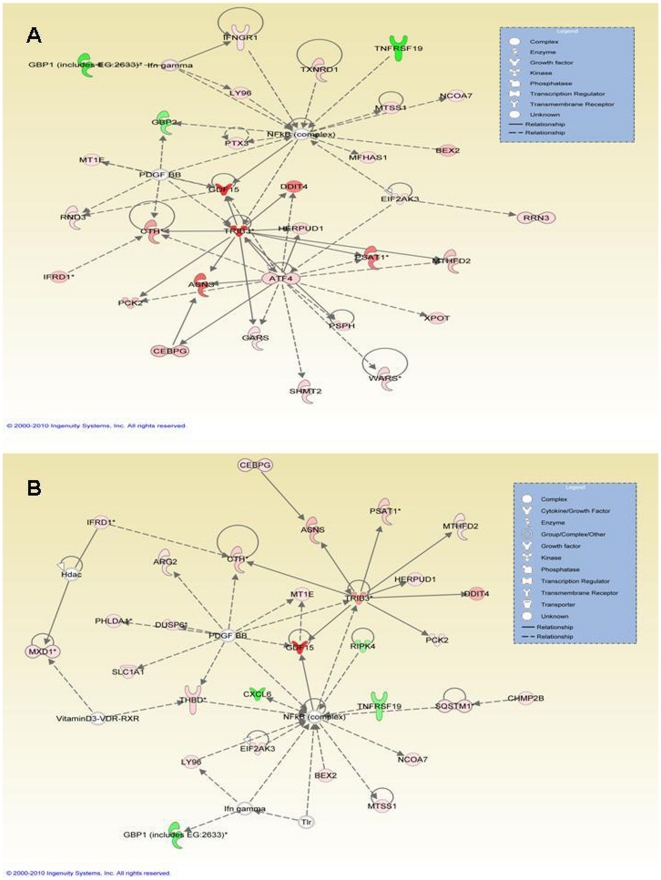
Functionally related gene networks constructed from the (A) normal untreated vs normal rottlerin-treated comparison or (B) SSc untreated vs SSc rottlerin-treated comparison. Differentially expressed genes for each comparison were entered into Ingenuity Pathwork Analysis (IPA) software v 5.0. Nodes represent genes, with their shape representative of the functional classification of the gene product as depicted in the inset box. All of the upregulated genes are displayed in red whereas all of the downregulated genes are displayed in green and the degree of differential expression is reflected by the intensity of the color, with darker colors indicating a greater level of differential expression.

To analyze the functional effects of the transcripts differentially regulated by rottlerin the entirety of differentially expressed transcripts in each comparison (normal untreated vs. normal rottlerin treated, and untreated SSc vs. rottlerin treated SSc) was loaded into the pathway analysis program in order to generate network, functional, and pathway analyses. The pathway analysis converted the lists of Affymetrix ID for transcripts with accompanying expression level information into a set of relevant networks based on the Ingenuity Pathways Knowledge Base (IPKB). The transcripts were categorized based upon molecular function in the IPA software and the identified transcripts were also mapped to genetic networks in the IPA database and ranked by score. This score reflects the probability that a collection of genes equal to or greater than the number in a particular network could be achieved by chance alone. A score of greater than 25 was used as a cutoff for identifying gene networks. The network with the highest score for the normal untreated control vs. normal rottlerin treated comparison is composed of 32 differentially regulated genes and is depicted in **Figure 3a**. The network with the highest score for the untreated SSc versus the rottlerin treated SSc comparison is composed of 29 differentially regulated genes and is depicted in **Figure 3b**. The networks for each comparison share 19 genes in common with each other, whereas the network generated in the untreated normal versus the rottlerin treated comparison contains 13 unique genes and the network generated in the untreated SSc versus the rottlerin treated SSc comparison contains 10 unique genes. Interestingly, the transcription factor NFκB appears as a central molecule in both networks even though its transcription levels are not directly affected. The list of networks generated for the untreated normal vs. rottlerin treated normal comparison is displayed in **Table 4** and the list of networks generated for the comparison between untreated SSc and rottlerin treated SSc is displayed in **Table 5**.

**Table 4:** Selected genetic networks with high scores (>26) in Normal Control vs Normal Rottlerin-exposed human dermal fibroblasts.

Molecules in Network	Score	Focus Genes	Top Functions
ASNS↑, ATF4↑, BEX2↑, CEBPG↑, CTH↑, DDIT4↑, EIF2AK3↑, GARS↑, GBP2↓, GBP1 ↓, GDF15↑, HERPUD1↑, IFN-γ↑, IFNGR1↑, IFRD1↑, LY96↑, MFHAS1↑, MT1E↑, MTHFD2↑, MTSS1↑, NCOA7↑, NFkB (comp), PCK2↑, PDGF BB, PSAT1↑, PSPH↑, PTX3↑, RND3↑, RRN3↑, SHMT2↑, TNFRSF19↓, TRIB3↑, TXNRD1↑, WARS↑, XPOT↑	64	32	Amino acid metabolism; small molecule biochemistry; carbohydrate metabolism
AKT, APC, BIRC5↓, C1ORF103↑, C5ORF13↓, Caspase, CDC20↓, CDCA8↓, Cyclin A, Cyclin E, DHFR↓, E2f, FGF2↑, GPNMB↑, Hdac, Histone h3, Histone h4, HMMR↓, Hsp27↓, Hsp70↑, HSPB1↓, MCM5↓, MKI67↓, MT1F↑, MT1G↑, MXD1↑, NUPR1↑, NUSAP1↓, PTGS1↑, Rb, Rsk, TGIF1↑, TPX2↓, TYMS↓, VEGFA↑	37	22	Cell cycle; cardiovascular system development and function; organismal development
CARHSP1↓, CENPE, CHMP2B, COG5 (includes EG:10466) ↑, DSN1, ELK3, ERBB2, GTSE1↓, HSPA9↑, JMY↑, Jmy-p300↑, KIAA0101↓, MIS12, MKI67↓, MXD3↓, NBR1, NCAPD2, NCAPG (includes EG:64151) ↓, NCAPH, NDC80↓, NUF2↓, POLG, POLH↓, PSRC1↓, PXN, RPSA, SH2D5↑, SLC19A2↑, SPC24, SPC25↓, SQSTM1↑, TACC3↓, TP53, TTC5, ZWINT (includes EG:11130) ↓	29	19	Cell cycle; cellular assembly and organization; DNA replication, recombination and repair
ABCG8, ACOX1, ACSL1, ARG2, BAAT, BNC1↑, CDCA2↓, DNMT3L, EXO1↓, FABP2, FAM65B↓, GPT2↑, HDAC1, HNF4A, HUNK↓, MDH1, MOCOS↑, NAGA, NDUFA1, NDUFV1, NRBF2↑, NUCB1, PCK2↑, PPP1CA, PSMA3, RSL24D1↑, RXRA, SESN2↑, SLC2A4, SLC38A1↑, SLMO2↑, SYTL2↓, TCF19↓, TUBE1↑, UHRF1BP1↑	26	17	Lipid metabolism; small molecule biochemistry; cardiovascular disease
ASB1↑, BCAT1↑, C9ORF72↑, CARS↑, CDCA3↓, CIDEC, EAF2↑, EPRS↑, GARS↑, HSP90AA1, IARS↑, IKBKG, Integrin alpha V beta 3↑, KCNG1↑, KDM5B, MARS↑, NCAPH, NDRG4↓, NET1, NFIL3, NOD2, PA2G4, PIR↑, PPAP2B, progesterone, PTGER4, RYR3, SAA1, SLC39A14↑, SMYD3↑, SRXN1↑, TGFB1, TMEM14A↓, TNFSF11, ZFP36	26	17	Cellular growth and proliferation; embryonic development; reproductive system function and development

**Table 5:** Selected genetic networks with high scores (>26) in SSc Control vs SSc Rottlerin-exposed human dermal fibroblasts.

Molecules in Network	Score	Focus Genes	Top Functions
ARG2↑, ASNS↑, BEX2↑, CEBPG↑, CHMP2B↑, CTH↑, CXCL6↓, DDIT4↑, DUSP6↑, EIF2AK3↑, GBP1 (includes EG:2633) ↓, GDF15↑, Hdac, HERPUD1↑, Ifn gamma, IFRD1↑, LY96↑, MT1E↑, MTHFD2↑, MTSS1↑, MXD1↑, NCOA7↑, NFkB (complex), PCK2↑, PDGF BB, PHLDA1↑, PSAT1↑, RIPK4↓, SLC1A1↑, SQSTM1↑, THBD↑, Tlr, TNFRSF19↓, TRIB3↑, VitaminD3-VDR-RXR	59	29	Amino acid metabolism; small molecule biochemistry; genetic disease
CBS↑, DBP, DCK, ELL, GAS1↓, GDF15↑, GINS2↓, GINS4↓, GSTM4, HDAC8, HNF4A, HSPH1, HUNK↓, JMY↑, Jmy-p300↑, KLHL24↑, MIR124, MOCOS↑, NAMPT↑, NBR1, RCHY1, RMND1, RSL24D1↑, SEL1L, SESN2↑, SLC17A5↑, SLC31A1↑, SLC38A1↑, SNAI2, TEAD2↓, TP53, TRIP11, TTC5, TUBE1↑, UBQLN2	32	19	Cell-to-cell signaling and interaction; cellular function and maintenance; infection mechanism
Collagen(s), CXCL12↓, Elastase, ERK, Fibrin, Fibrinogen, FN1↓, Focal adhesion kinase, GFPT1↑, HOMER1↑, ICAM1↑, IL8↑, Integrin, Integrin alpha 3 beta 1, LDL, LRP, Mek, MKNK2↑, NAMPT↑, NfkB1-RelA, NPC1↑, NRP2↑, Pak, Pdgf, PLAUR↑, Rac, Rap1, RCAN1↑, SLC3A2↑, SLC7A5↑, SLC7A11↑, THBS1↓, Vegf↑, VEGFA↑, VLDLR↑	29	18	Cellular assembly and organization; cellular function and maintenance; protein trafficking
Ap1, ARL4C↑, ASF1B↓, ATP2B1↑, Creb, Cyclin A, DNAJB9↑, E2f, EPOR↑, ERK1/2, FSH, G alphai, GAS1↓, GOT1↑, hCG, Histone h3, Histone h4, IL12 (complex), JARID2↑, Lh, Mapk, MCM4↓, MCM5↓, MCM10↓, MT1F↑, MT1X↑, PFKFB2↑, Pka, Pkc(s), PLC, PLC gamma, Pld↑, PP2A, STX3↑, UHRF1↓	26	16	Hematological system development and function; hematopoiesis; tissue morphology
Akt, C1ORF103↑, Caspase, CLIP1↑, Cyclooxygenase↑, EIF4EBP1↑, FTH1↑, G-protein beta, GADD45A↑, GNA13↑, Gpcr, HMOX1↑, Hsp27↓, Hsp70↑, HSPA5↑, HSPA9↑, HSPB3↓, IFN Beta, IKK (complex), IL1, Insulin, Interferon alpha, Jnk, MHC Class II, NUPR1↑, P38 MAPK, PI3K, PTGS1↑, PTGS2↑, Ras, Ras homolog↓, RORA↑, Sapk, TGIF1↑, WARS↑	26	16	Cardiovascular system development and function; connective tissue disorders; drug metabolism

The functional pathway analysis demonstrated that PKC-δ inhibition affected the transcriptional levels of genes from diverse pathways that are involved in a variety of pathological conditions, such as: cancer, inflammatory, gastrointestinal, reproductive, cardiovascular and hematological diseases. As reported in numerous studies, PKC-δ inhibition differentially regulated genes involved in diverse cellular functions including cell death, cell cycle control, growth and differentiation, amino acid metabolism, small molecule biosynthesis, cell to cell signaling and interaction, as well as, cellular movement [8]–[12]. Numerous genes involved in organismal development and tumor morphology were also regulated.

### Targeting of PKC-δ activity using RNA interference and a PKC-δ inhibitory cell-permeable peptide

Although nanomolar concentrations of rottlerin potently inhibit PKC-δ activity, at higher concentratiions rottlerin can also suppress the activity of other kinases [25] and can also uncouple mitochondrial respiration from oxidative phosphorylation. To ensure that the observed effect of rottlerin on the transcriptome is due to its inhibition of PKC-δ and is not the result of possible effects on other protein kinases or caused by rottlerin-induced mitochondrial uncoupling, PKCδ activity was also targeted in fibroblasts via RNA interference and the use of a specific cell permeable PKC-δ inhibitory peptide. Normal and SSc fibroblasts were transfected with control and PKC-δ specific siRNA or were cultured in media containing either a control peptide consisting of a dimer of the TAT protein transduction domain (PTD) [21] or with a PKC-δ specific inhibitory peptide that targets the N-terminal C2 domain fused to a single TAT PTD [22]. The C2 domain of PKC isoenzymes is a region within the regulatory domain that mediates protein-protein interactions between individual PKC isozymes and their achoring proteins, receptors for activated C kinase (RACKs). The PKC-δ -specific peptide binds to the specific RACKs, preventing PKC-δ binding, thereby disrupting the anchoring and functioning of PKC-δ [26]. RNA from these experiments was utilized in validation of gene expression regulated by PKC-δ inhibition. The effects of rottlerin, of RNA interference and of peptide-mediated PKC-δ inhibition on the expression of PKC-δ in normal and SSc fibroblasts were evaluated by real time RT-PCR. The PKC-δ specific siRNA induced an 84% decrease in PKC-δ mRNA levels in normal fibroblasts and an 88% decrease in SSc cells (data not shown). The control siRNA had no appreciable effect on PKC-δ expression compared to the saline control. As expected, the PKC-δ inhibitory peptide did not induce a change in PKC-δ mRNA levels in either normal or SSc fibroblasts.

### Validation of genes differentially expressed in response to PKC-δ inhibition by real time RT-PCR

**Figure 4:**
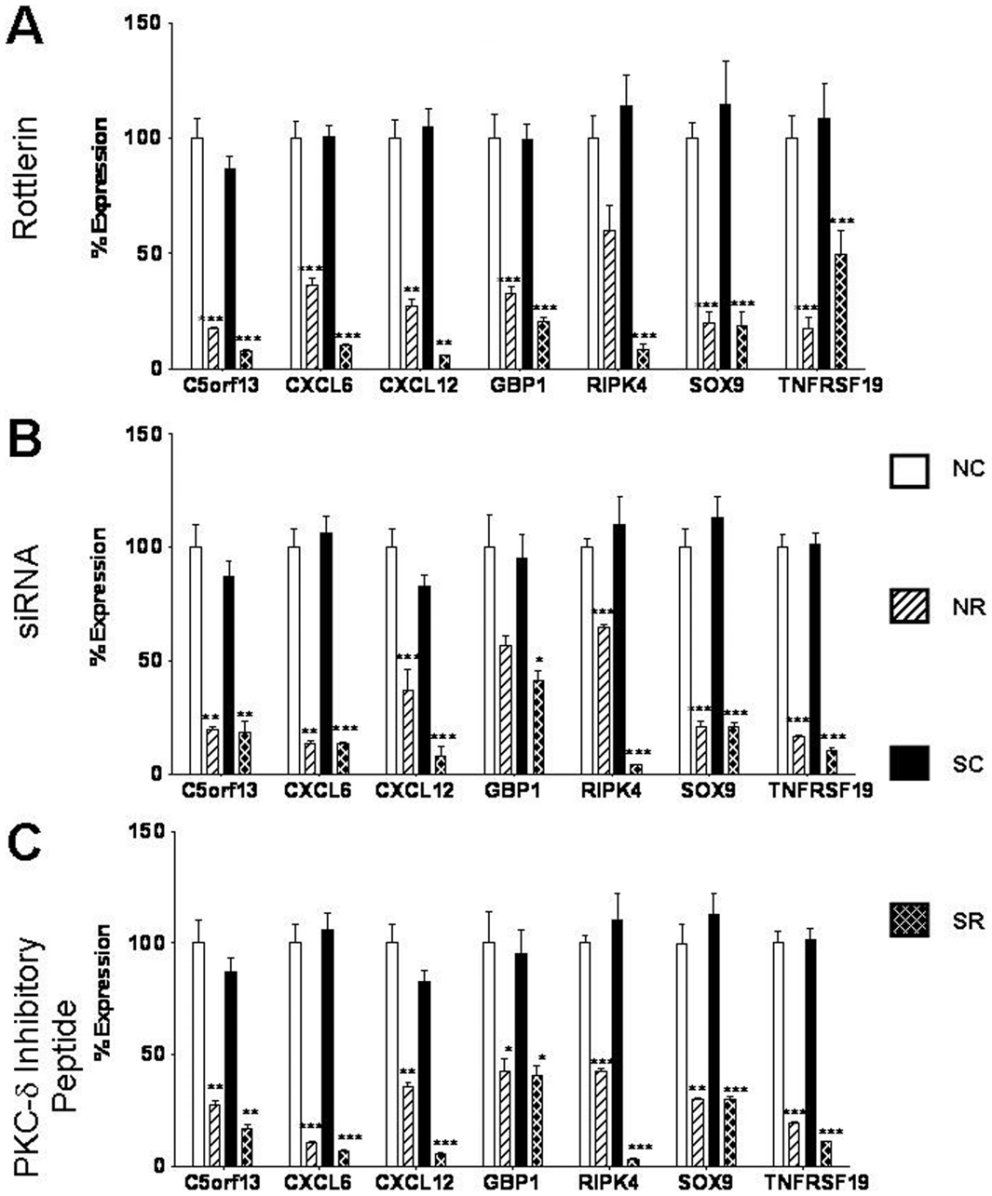
Validation of expression levels of downregulated genes following PKC-δ inhibition. **A.** mRNA expression levels of 7 downregulated genes in normal and SSc human dermal fibroblasts following treatment with **A.** 5 µM rottlerin, **B.** 10 nM PKC-δ siRNA, **C.** 10 µM cell permeable specific PKC-δ inhibitory peptide for 24 h. Results are expressed as mean percent difference +/− SD of 3 replicate samples analyzed by quantitative RT-PCR. The PBS control was arbitrarily set to 100% expression. NC: Normal untreated fibroblasts, NR: Normal treated fibroblasts, SC: SSc untreated fibroblasts, SR: SSc treated fibroblasts. Statistical significance was calculated to compare treated vs untreated normal or SSc fibroblasts. *: p<0.1; **: p<0.01; ***: p<0.0001.

The relative expression levels of selected highly expressed genes and of genes from the two most significant networks obtained in the IPA analysis were confirmed by real time RT-PCR performed on RNA isolated from each of three sources: rottlerin-treated fibroblasts, siRNA-treated fibroblasts or peptide-treated fibroblasts. The successful validation of a total of 12 genes is displayed in **Figures 4** **and** **5**. Seven genes that were downregulated in response to rottlerin were selected for validation. These were: chromosome 5 open reading frame 13 (c5orf13), chemokine CXC motif ligand 6 (CXCL6), chemokine CXCL12, interferon-inducible guanylate binding protein 1 (GBP1), receptor interacting serine/threonine kinase 4 (RIPK4), also known as PKC-δ interacting protein kinase (DIK) or ankyrin repeat domain-containing protein kinase 3 (ANKRD3); SRY-box 9 (SOX9) and tumor necrosis factor receptor superfamily, member 19 (TNFRSF19). **Figure 4a** displays the real time RT-PCR expression levels for these 7 downregulated genes in rottlerin treated fibroblasts, whereas **Figure 4b** displays expression levels for the same set of genes in siRNA-treated fibroblasts and **Figure 4c** shows the expression of these genes in fibroblasts treated with the PKC-δ inhibitory peptide. The control siRNA and the control peptide did not affect the expression levels of these genes compared to the saline control. All genes examined demonstrated the same consistent pattern of decreased expression in response to PKC-δ inhibition that was observed in the microarray analysis.

**Figure 5:**
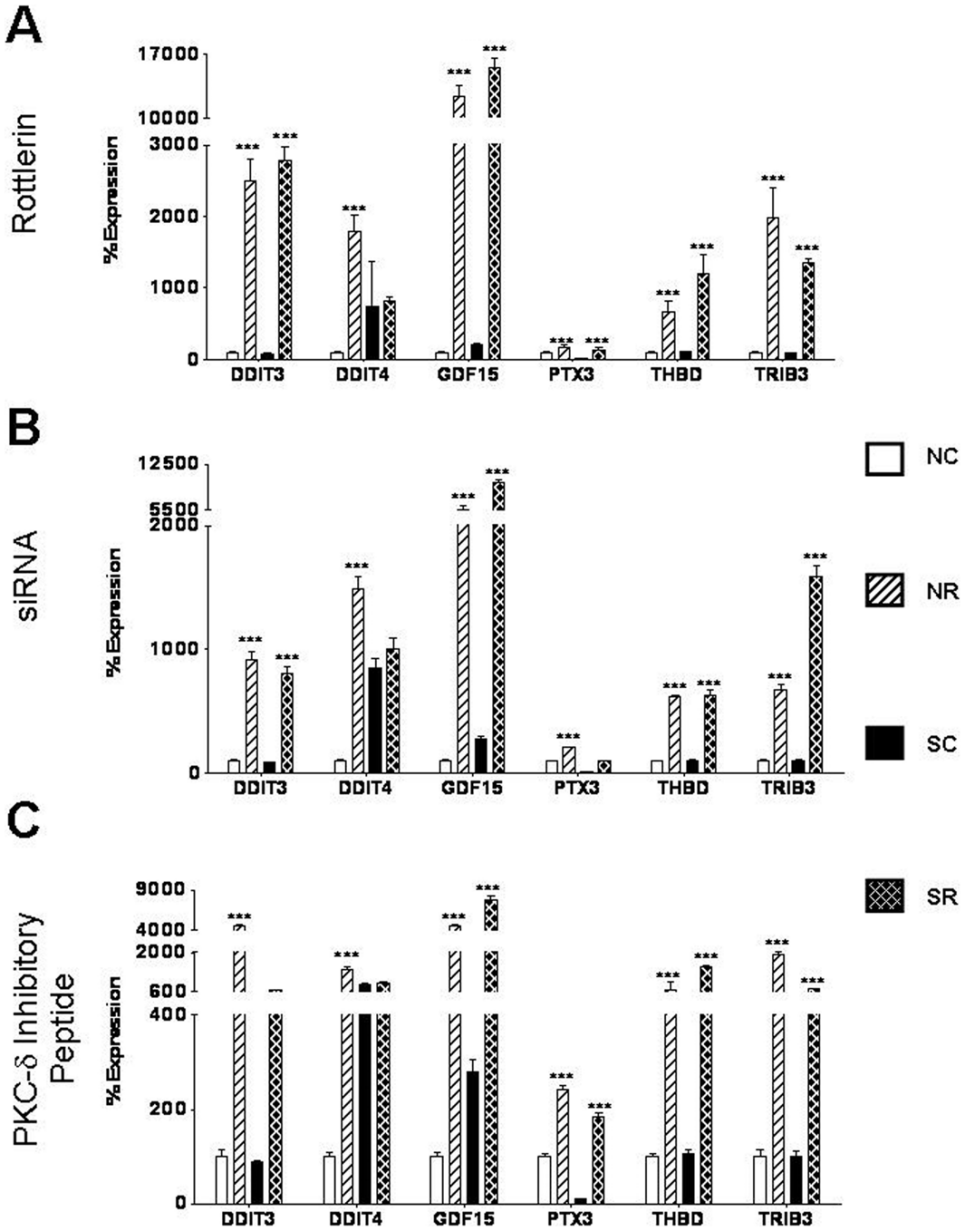
Validation of expression levels of upregulated genes following PKC-δ inhibition. **A.** mRNA expression levels of 6 upregulated genes in normal and SSc human dermal fibroblasts following treatment with **A.** 5 µM rottlerin, **B.** 10 nM PKC-δ siRNA, **C.** 10 µM cell permeable specific PKC-δ inhibitory peptide for 24 h. Results are expressed as mean percent difference +/− SD of 3 replicate samples analyzed by quantitative RT-PCR. The PBS control was arbitrarily set to 100% expression. NC: Normal untreated fibroblasts, NR: Normal treated fibroblasts, SC: SSc untreated fibroblasts, SR: SSc treated fibroblasts. Statistical significance was calculated to compare treated vs untreated normal or SSc fibroblasts. *: p<0.1; **: p<0.01; ***: p<0.0001.

Six genes that were upregulated in response to PKC-δ inhibition were also chosen for validation. These were, DNA damage inducible transcript 3/C/EBP homologous protein (DDIT3/CHOP), DNA damage inducible transcript 4/Regulated in development and DNA damage responses 1 (DDIT4/REDD1), growth/differentiation factor 15 (GDF15), pentraxin 3 (PTX3) thrombomodulin (THBD), and tribbles homolog 3 (TRIB3). **Figure 5a** displays the real time RT-PCR expression levels for these 6 upregulated genes in rottlerin treated fibroblasts, whereas **Figure 5b** displays expression levels for the same set of genes in siRNA-treated fibroblasts and **Figure 5c** shows the expression of these genes in fibroblasts treated with the PKC-δ inhibitory peptide. The control siRNA and the control peptide did not affect the expression levels of these genes compared to the saline control. All genes examined displayed changes in expression in cells in which PKC- δ was inhibited by each of three methods consistent with those observed in the microarray analysis.

### Genes differentially regulated in SSc-derived dermal fibroblasts

A similar analysis of differentially expressed transcripts between untreated normal fibroblasts versus untreated SSc-derived fibroblasts (normal control vs. SSc control) and between rottlerin treated normal fibroblasts versus rottlerin treated SSc-derived fibroblasts (normal rottlerin vs. SSc rottlerin) yielded a total of 75 genes that displayed a 2 fold or greater difference in expression at a p-value of p<0.05 (**Table 1**). Since SSc is a complex disorder and multiple studies of the SSc transcriptome have reported wide variability in expression patterns, the criteria for analysis were broadened to include transcripts displaying a 1.5 fold or greater change in expression level with a p-value of 0.10. This resulted in the identification of a total of 360 differentially expressed genes (191 upregulated and 169 downregulated) between normal versus SSc-derived fibroblasts irrespective of whether or not the cells had been treated with rottlerin (**Table 1**). Of these 360 genes, 61 genes were differentially expressed (38 upregulated and 23 downregulated) in both the untreated and rottlerin-treated SSc fibroblasts compared to their normal counterparts. Untreated fibroblasts demonstrated 134 transcripts (62 upregulated and 72 downregulated) that were differentially regulated in SSc-derived fibroblasts compared to normal-derived fibroblasts, whereas rottlerin treated SSc-derived fibroblasts displayed 165 unique transcripts (91 upregulated and 74 downregulated) that were differentially regulated compared to normal-derived fibroblasts. A partial list of the most upregulated transcripts in SSc fibroblasts is displayed in **Table 6** and a partial list of the most downregulated transcripts in SSc fibroblasts is displayed in **Table 7**.

**Table 6:** Selected transcripts upregulated in SSc fibroblasts.

Gene Symbol	Description	Fold Change
**Transcripts Upregulated in SSc vs NormalFibroblasts**		
IGFBP5	insulin-like growth factor binding protein 5	5.0
FGFR2	fibroblast growth factor receptor 2	4.6
POMZP3	POM and ZP3 fusion zona pellucida glycoprotein 3	4.5
FGFR2	fibroblast growth factor receptor 2	4.4
FGFR2	fibroblast growth factor receptor 2	4.3
POMZP3	POM and ZP3 fusion zona pellucida glycoprotein 3	4.1
CADP5	Ca++-dependent secretion activator 5	3.9
NFIB	nuclear factor I/B	3.8
MCAM	melanoma cell adhesion molecule	3.3
MRVI1	murine retrovirus integration site 1	3.2
**Transcripts Upregulated in Untreated SSc vs Untreated Normal FibroblastsCells Only**		
ACTG2	actin, gamma 2, smooth muscle, enteric	6.0
ITGA7	integrin, alpha 7	4.4
ANK2	ankyrin 2, neuronal	4.4
CLIC3	chloride intracellular channel 3	4.3
EBF1	early B cell factor 1	4.3
ANK2	ankyrin 2, neuronal	4.2
CDO1	cysteine dioxygenase 1	4.2
HSPB7	heat shock 27 kDa protein family, member 7	3.8
AFF3	AF4/FMR2 family, member 3	3.6
CAMK2D	calcium/calmodulin-dependent protein kinase II delta	3.4
CD74	CD74 molecule, MHC, class II invariant chain	3.4
**Transcripts Upregulated in SSc Rottlerin-Treated vs Normal Rottlerin-Treated Fibroblasts Cells Only**		
DES	desmin	5.6
PRUNE2	prune homolog 2 (Drosophila)	5.3
CSGALNA	chondroitin sulfate Nacetylgalactosaminyltransferase 1	5.2
ACTC1	actin, alpha, cardiac muscle 1	5.0
TM4SF20	transmembrane 4 L six family member 20	3.8
PLAU	plasminogen activator, urokinase	3.7
FHL1	four and a half LIM domains 1	3.6
FOS	v-fos FBJ murine osteosarcoma viral oncogene	3.6
FHL1	four and a half LIM domains 1	3.3
FHL1	four and a half LIM domains 1	3.2
MEF2C	myocyte enhancer factor 2C	3.1

Fold change indicates the difference between untreated or treated normal cells compared to untreated or treated SSc cells. The entire dataset discussed in this paper is deposited at Gene Expression Omnibus (http://www.ncbi.nlm.nih.gov/geo/query/acc.cgi?acc=GSE23741) under accession number GSE23741.

**Table 7:** Selected transcripts downregulated in SSc fibroblasts.

Gene Symbol	Description	Fold Change
**Transcripts Downregulated in SSc vs NormalFibroblasts**		
CDH2	cadherin 2, type 1, neuronal	5.0
B3GACTL	beta 1,3-galactosyltransferase-like	4.6
GRIK2	glutamate receptor, ionotropic, kainate 2	4.5
SSTR1	somatostatin receptor 1	4.4
DACT1	dapper, antagonist of beta-catenin, homolog 1	4.3
CDH2	cadherin 2, type 1, neuronal	4.1
TSPAN13	tetraspanin 13	3.9
SIPR1	sphingosine-1-phosphate receptor 1	3.8
TBX3	T-box 3	3.3
NID2	nidogen 2 (osteonidogen)	3.2
COL13A1	collagen, type XIII, alpha 1	2.9
**Transcripts Downregulated in Untreated SSc vs Untreated Normal FibroblastsCells Only**		
DAB1	disabled homolog 1 (Drosophila)	6.0
PTGS1	prostaglandin-endoperoxide synthase 2	4.4
TMTC2	transmembrane and tetratricopeptide repeat cont. 2	4.4
RUNX3	runt-related transcription factor 3	4.3
PID1	phosphotyrosine interaction domain containing 1	4.3
SOCS2	suppressor of cytokine signaling 2	4.2
CRISPLD2	cysteine-rich secretory protein LCCL domain cont. 2	4.2
IFI27	interferon, alpha-inducible protein 27	3.8
CDKN1C	Cyclin-dependent kinase inhibitor 1C (p57, Kip2)	3.6
DSEL	dermatan sulfate epimerase-like	3.4
NDRG4	NDRG family member 4	3.4
**Transcripts Downregulated in SSc Rottlerin-Treated vs Normal Rottlerin-Treated Fibroblasts Cells Only**		
LHX8	LIM homeobox 8	8.2
MMP3	matrix metallopeptidase 3 (stromelysin 1)	5.3
C9orf167	chromosome 9 open reading frame 167	3.6
AKR1C1	Aldo-keto reductase family 1, member C1	3.5
C9orf167	chromosome 9 open reading frame 167	3.0
CALB2	calbindin 2	2.8
FOXF1	forkhead box F1	2.7
DOK6	docking protein 6	2.6
DIO2	deiodinase, iodothyronine, type II	2.6
MALAT1	metastasis assoc. lung adenocarcinoma trans. 1	2.5
FAM44A	family with sequence similarity 44, member A	2.4

Fold change indicates the difference between untreated or treated normal cells compared to untreated or treated SSc cells. The entire dataset discussed in this paper is deposited at Gene Expression Omnibus (http://www.ncbi.nlm.nih.gov/geo/query/acc.cgi?acc=GSE23741) under accession number GSE23741.

**Figure 6:**
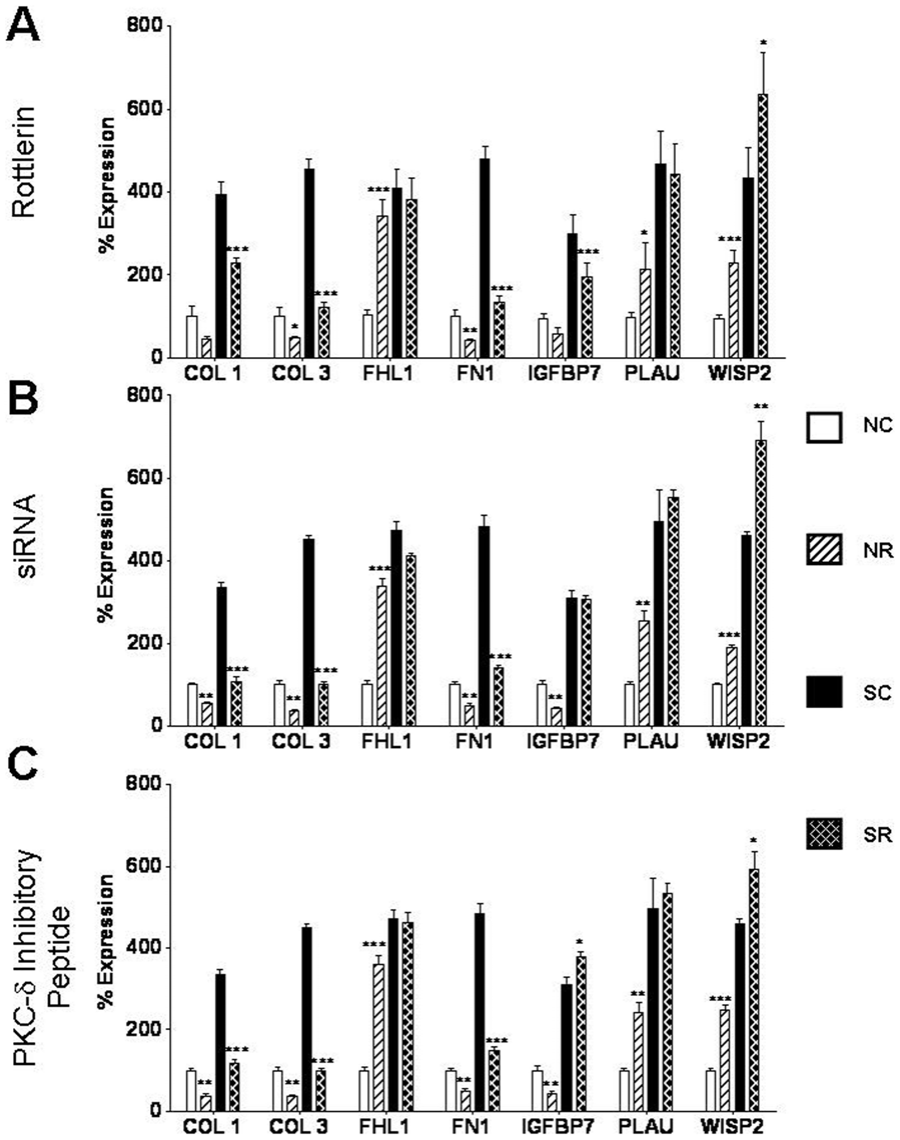
Validation of expression levels of genes upregulated in SSc fibroblasts compared to normal fibroblasts following PKC-δ inhibition. mRNA expression levels of 7 upregulated genes in normal and SSc human dermal fibroblasts following treatment with **A.** 5 µM rottlerin, **B.** 10 nM PKC-δ siRNA, **C.** 10 µM cell permeable specific PKC-δ inhibitory peptide for 24 h. Results are expressed as mean percent difference +/− SD of 3 replicate samples analyzed by quantitative RT-PCR. The PBS control was arbitrarily set to 100% expression. NC: Normal untreated fibroblasts, NR: Normal treated fibroblasts, SC: SSc untreated fibroblasts, SR: SSc treated fibroblasts. Statistical significance was calculated to compare treated vs untreated normal or SSc fibroblasts. *: p<0.1; **: p<0.01; ***: p<0.0001.

Several genes known to be involved in the regulation of production and maintenance of the extracellular matrix (ECM) or in the development of fibrosis, including fibronectin 1 (FN1), insulin growth factor binding protein 7 (IGFBP7), plasminogen activator urokinase (PLAU), Wnt-inducible signaling protein 2 (WISP2), were upregulated in SSc-derived dermal fibroblasts compared to normal dermal fibroblasts. The expression patterns of transcripts for these genes in control and PKC- δ-inhibited normal and SSc fibroblasts, as well as for those encoding type I and type III collagen were examined by real time RT-PCR. **Figure 6a** displays expression levels in rottlerin treated normal and SSc fibroblasts whereas **Figure 6b** displays expression levels in siRNA treated fibroblasts and **Figure 6c** displays expression of these genes in fibroblasts treated with the inhibitory PKC-δ peptide. The control siRNA and the control peptide did not affect the expression levels of these genes compared to the saline control. Expression of these genes as measured by real time PCR was consistent with the pattern observed in the microarray results.

**Figure 7:**
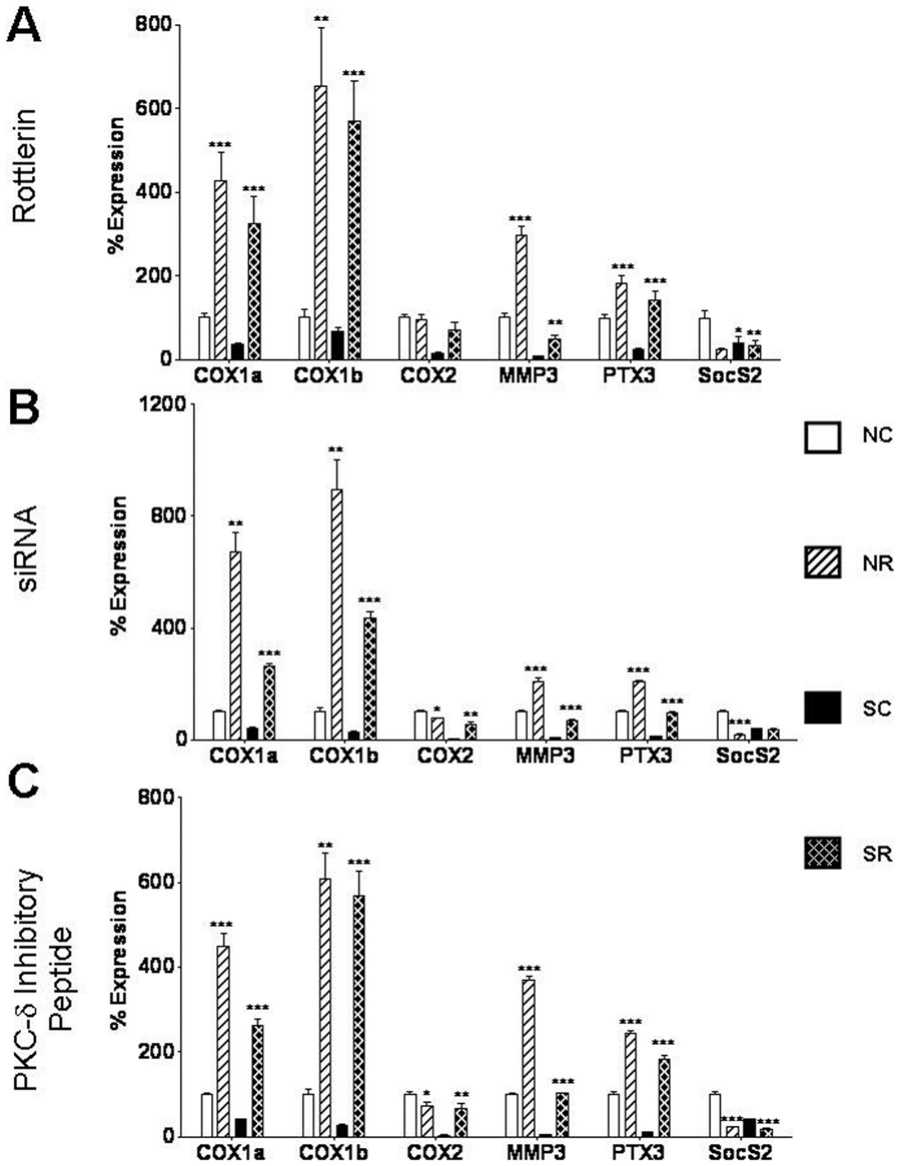
Validation of expression levels of genes downregulated in SSc fibroblasts compared to normal fibroblasts following PKC-δ inhibition. mRNA expression levels of 6 downregulated genes in normal and SSc human dermal fibroblasts following treatment with **A.** 5 µM rottlerin, **B.** 10 nM PKC-δ siRNA, **C.** 10 µM cell permeable specific PKC-δ inhibitory peptide for 24 h. Results are expressed as mean percent difference +/− SD of 3 replicate samples analyzed by quantitative RT-PCR. The PBS control was arbitrarily set to 100% expression. NC: Normal untreated fibroblasts, NR: Normal treated fibroblasts, SC: SSc untreated fibroblasts, SR: SSc treated fibroblasts. Statistical significance was calculated to compare treated vs untreated normal or SSc fibroblasts. *: p<0.1; **: p<0.01; ***: p<0.0001.

Microarray results indicated that cyclooxgenase 1/prostaglandin synthase 1 (COX1/PTGS1) and cyclooxygenase 2/prostaglandin synthase 2 (COX2/PTGS2), important mediators of arachidonic acid and prostaglandin synthesis were strongly downregulated in untreated SSc-derived fibroblasts compared to normal untreated fibroblasts as were matrix metalloproteinase 3 (MMP3), pentraxin-3 (PTX3) and suppressor of cytokine signaling 2 (SOCS2). Rottlerin strongly induced upregulated expression COX1/PTGS1, MMP3 and PTX3 but upregulated expression of COX2/PTGS2 was observed only in rottlerin-treated SSc fibroblasts and upregulated SocS2 expression only in rottlerin-treated normal fibroblasts The expression patterns of transcripts for these genes in control and PKC- δ-inhibited normal and SSc fibroblasts, as well as for those encoding type I and type III collagen were examined by real time RT-PCR. **Figure 7a** displays expression levels in rottlerin treated normal and SSc-derived fibroblasts whereas **Figure 7b** displays expression levels in siRNA treated fibroblasts and **Figure 7c** displays expression of these genes in fibroblasts treated with the inhibitory PKC- δ peptide. The control siRNA and the control peptide did not affect the expression levels of these genes compared to the saline control. COX1a/PTGS1a, COX1b/PTGS1b, COX2/PTGS2, MMP3, PTX3 and SOCS2 were downregulated in untreated SSc fibroblasts versus untreated normal fibroblasts (**Figure 7a–c**). PKC-δ inhibition strongly induced expression of COX1a/PTGS1a, COX1b/PTGS1b, MMP3 and PTX3 in both normal and SSc fibroblasts whereas COX2/PTGS2 expression was induced only in treated SSc fibroblasts and SocS2 expression was induced only in treated normal fibroblasts. This expression pattern was consistent with the pattern observed in the microarray results. Interestingly, treatment of SSc fibroblasts with rottlerin, siRNA or PKC- δ-inhibitory peptide increased the expression levels of COX1 isoforms 1 and 2, COX2, MMP3 and PTX3 (**Figure 7a–c**). Expression of type I and type III collagen were also upregulated in SSc-derived fibroblasts compared to normal fibroblasts by real time RT-PCR although this upregulation was not observed in the microarray results, possibly due to the very high fibroblast expression levels of these genes.

To further confirm the validity of the results, the production of type I collagen by the cultured fibroblasts and the effect of rottlerin on type I collagen production by these cells was evaluated by Western blots of culture supernatants. Consistent with previously described results, SSc-derived dermal fibroblasts showed substantially increased levels of type I collagen production compared to normal dermal fibroblasts. Exposure to rottlerin induced a dramatic reduction in type I collagen production in both normal and SSc-derived dermal fibroblasts (data not shown).

### Analysis of common transcription regulatory networks

Analysis of the promoter regions of the 443 genes differentially regulated in response to rottlerin was performed by entering the transcript IDs into the PAINT program. The analysis revealed a statistically significant association between the presence of specific TREs and exposure to rottlerin. The results of promoter analysis filtered by p<0.05 with the FDR set at <0.3 demonstrated 14 transcription factors whose TREs were significantly enriched in response to rottlerin. These factors were: transcription factor CP2 (TFCP2), olfactory neuronyl transcription factor 1/early B-cell factor 1 (OLF1/EBF1), aryl hydrocarbon receptor nuclear translocator/hypoxia inducible factor 1 (ARNT/HIF1), myogenin (MYOG), NFκB, E2F, selenocysteine tRNA gene transcription activating factor (STAF), hepatocyte nuclear factor 4 (HNF4), paired box gene 6 (PAX6), CCAAT box binding protein, cooperates with myogenic protein 1 (COMP1), upstream stimulatory factor (USF), v-ets avian erythroblastosis virus E26 oncogene homolog 1(c-ETS1) and E26-like protein 1 (ELK1) as shown in **Table S2**. The identification of statistically significant enrichment of a specific TRE within a particular expression cluster may indicate a role for the cognate transcription factor in coordinate regulation of genes in that cluster. An analysis of the 359 transcripts differentially regulated between normal and SSc-derived dermal fibroblasts identified 4 transcription factors TREs that were enriched in response to rottlerin. These factors were upstream transcription factor 1 (USF1), STAT1, cAMP response element binding protein (CREB) and cJun (**Table S2**).

## Discussion

PKC-δ has been implicated as a regulator of a wide variety of cellular processes and has been shown to participate in the pathogenesis of numerous disorders. PKC-δ also plays an important role in tissue fibrosis as it is regulated by TGF-β. TGF-β activates PKC-δ which in turn positively regulates Smad3 transcriptional activity, resulting in increased transcription of genes encoding various collagens and fibronectin [17], [18]. Other studies have shown that production and secretion of type I collagen by vascular smooth muscle cells requires PKC-δ [15]. Furthermore, PKC-δ activation is necessary to mediate the stimulatory effect of CTGF in cooperation with insulin/insulin growth factor 1 (IGF1) on collagen synthesis in SSc fibroblasts [14].

In a previous study we demonstrated that SSc fibroblasts have substantially increased levels of PKC-δ compared to normal fibroblasts and that exposure of these fibroblasts to rottlerin resulted in a >80% reduction in COL1A1 mRNA, and a >70% reduction in COL3A1 mRNA with a corresponding decrease in the production of these proteins. The role of PKC-δ in the regulation of collagen gene expression was further documented by the demonstration that a dominant-negative form of PKC-δ caused a potent decrease in the transcription of the type I collagen gene promoter [13].

Given the demonstrated effect of rottlerin to downregulate the production of type1 and type 3 collagens, we undertook the present study in order to gain a better understanding of the effects of rottlerin on the transcriptome of fibroblasts to identify molecules and pathways that may be important in the pathogenesis of fibrotic diseases such as SSc. Microarray analysis on total RNA isolated from rottlerin-treated and non-treated normal and SSc fibroblasts yielded a total number of 433 gene transcripts that displayed a greater than 2 fold difference in expression at a significance level of p<0.05 (**Table 1** **,** **Figure 1**). Since experimental replicates were not used in this study, transcripts displaying a fold change of less than 2 fold were excluded. The differentially regulated genes are involved in many of the processes already attributed to be regulated by PKC-δ activity. These include: cell death, cell cycle control, cell growth and proliferation, cellular movement, cell-cell interaction, amino acid synthesis and tumor morphology as well as embryonic, tissue, hematopoietic and skeletal development. The differentially regulated genes also are associated with the development of a variety of disease conditions, such as: cancer, gastrointestinal, reproduction, cardiovascular and hematological diseases as well as various genetic disorders. The gene network displaying the greatest number of genes that were differentially regulated showed that one of the central molecular hubs was the NFκB transcription factor. The important role of NFκB was an observation strongly supported by the PAINT analysis of the TREs that are enriched at a significantly greater level than chance in untreated versus rottlerin treated fibroblasts which identified the NFκB TRE.

Although several recent papers have shown that rottlerin is a potent PKC-δ inhibitor exerting its effects on PKC-δ at nanomolar concentrations, it has become apparent that at much higher concentrations it can also inhibit other kinases [25]. Rottlerin can also uncouple mitochondrial respiration from oxidative phosphorylation and some of the effects on apoptosis previously attributed to PKC-δ could be caused by mitochondrial uncoupling and increased ROS production. However, the results we obtained did not provide compelling evidence of a role in mitochondrial uncoupling in dermal fibroblasts, one of the effects attributed to rottlerin, although, on the contrary, three genes encoding proteins located in the mitochondria [27]–[29] were upregulated in response to rottlerin, namely, mitochondrial phosphoenolpyruvate carboxypeptidase 2 (PCK2), mitochondrial serine hydroxymethyltransferase 2 (SHMT2), and mitochondrial lon peptidase 1 (LONP1). Among these only LONP1 has been reported to be affected by mitochondrial uncoupling [29]. Additionally, in order to address the possibility that the microarray results reflected changes in the transcriptome mediated by targets of rottlerin other than PKC-δ, we also isolated and analyzed RNA from normal and SSc fibroblasts in which PKC-δ activity had been targeted by RNA interference or with a cell-permeable PKC-δ inhibitory peptide. The real time PCR results from these PKC-δ inhibited fibroblasts support the conclusion that the differential expression observed in the microarray data can be attributed to inhibition of PKC-δ activity by rottlerin.

Twelve genes that were part of the most differentially regulated networks (**Figure 3**) or that were among the most strongly differentially expressed genes identified in our microarray analysis were randomnly selected for verification by real time PCR. Changes in the expression levels of these genes were analyzed and confirmed using RNA from fibroblasts in which PKC-δ activity was targeted by exposure to rottlerin, exposure to PKC-δ inhibitory peptide or by RNA interferenece. The upregulated genes that were confirmed (**Figure 5**) were: DDIT3/CHOP, a dominant negative inhibitor of C/EBP and CCAAT/enhancer binding protein beta [30]; DDIT4/REDD1, a transcriptional target of p53 which is upregulated by hypoxia [31]; GDF15, also known as macrophage inhibitory cytokine 1 (MIC1) which is expressed in activated but not in resting macrophages [32]; THBD, an endothelial cell surface protein which forms a complex with thrombin and coverts it into a physiologic anticoagulant [33] and TRIB3, a negative regulator of the transcription factors AKT, NFκB, AP1 and oncogenic ras [34]. It was of interest that among the genes found to be downregulated by inhibition of PKC-δ activity there were several genes that are likely to participate in the development of fibrotic reactions. Thus, these genes represent novel or less well recognized participants in the fibrotic process that were upregulated by PKC-δ. The profibrogenic genes that were downregulated by PKC-δ inhibition (**Figure 4**), which were confirmed by RT-PCR, included c5orf13, which downregulates TGF-β1 and TGF-βR2 and has been shown to decrease collagen expression and induce differentiation of myofibroblasts to fibroblasts [35]; CXCL6, a chemokine that displays increased expression during progression of liver fibrosis [36]; CXCL12, a chemokine that is a powerful attractant for fibrocytes and that participates in the angiogenesis associated with chronic inflammation and fibrosis and has been found to be increased in pulmonary fibrosis tissues [37]–[39]; GBP1, an interferon inducible guanylate binding protein that is expressed at gap junctions and is involved in endothelial cell proliferation and invasion [40]; SOX9, which is coexpressed with Col2a1, the gene encoding type II collagen, the major cartilage matrix protein [41] and which has recently been suggested to participate in SSc fibrosis [42]; and TNFRSF19, which is expressed primarily in prostate cells and is capable of activating the JNK pathway and of inducing NFκB activation [43].

Analysis of the differential expression of genes between normal and SSc fibroblasts identified a total of 75 genes that displayed a greater than 2 fold difference in expression with a p value < 0.05. However, to make the results of our studies comparable to other previously published studies comparing the normal and SSc transcriptomes [44]–[47], we reanalyzed the data including genes with differential expression greater than 1.5 fold and a significance of p<0.10 to reflect the high level of heterogeneity known to exist even between fibroblasts derived from normal and SSc skin biopsies. Using these parameters, we identified 360 genes that were differentially expressed between normal and SSc fibroblasts. Interestingly, only 61 of these transcripts were unaffected by rottlerin treatment. However, 134 transcripts were differentially expressed only in untreated cells, while 165 transcripts were differentially expressed only in rottlerin treated cells.

Urokinase plasminogen activator (PLAU), which has been implicated as a suppressor of fibrosis via activation of MMPs [48] was upregulated in normal fibroblasts but not in SSc fibroblasts following PKC-δ inhibition (**Figure 6**). Expression of COX1a, COX1b, and COX2 which regulate prostaglandin synthesis thereby suppressing the synthesis of collagen [49], [50]; and SOCS2, which acts to suppress insulin growth factor 1 (IGF1) and growth hormone (GH) mediated deposition of excessive collagen [51] were significantly downregulated in untreated SSc cells (**Figure 7**). Treatment with rottlerin, PKC-δ specific siRNA or with a PKC-δ inhibitory peptide dramatically induced expression of COX1a, COX1b, COX2 and SOCS2, an effect which would be expected to abrogate some of the potent PKC-δ profibrotic effects. Furthermore, MMP3, a matrix metalloproteinase involved in collagen remodeling [52] and PTX3, a gene involved in innate immune responses that is found to be elevated in the serum of SSc patients and that is constitutively expressed in SSc fibroblasts [53], [54] were downregulated in SSc fibroblasts following inhibition of PKC-δ activity (**Figure 7**).

The general pattern observed for genes involved in tissue fibrosis is that those genes which act to induce fibrosis and stimulate collagen and ECM production were suppressed in fibroblasts exposed to rottlerin whereas those genes normally associated with the suppression of fibrosis, inhibition of collagen production or increased collagen degradation were upregulated in response to rottlerin treatment. These results indicate that the potent inhibitory effect of rottlerin treatment on collagen production that we previously reported is not due solely to a decrease in transcription of the type I collagen genes but, instead, it appears to be the result of an inhibitory effect on multiple genes involved in the upregulation of expression of profibrotic molecules and a stimulatory effect on genes encoding collagenolytic and other antifibrotic molecules. These observations, therefore, indicate a much broader participation of PKC-δ in the pathogenesis of tissue fibrosis and suggest that inhibition of the broad spectrum of its profibrogenic effects may be a novel and effective therapeutic approach for SSc and other fibrosing diseases.

## Supporting Information

Table S1 **Primers used for real-time PCR studies.** PCR primers used to validate real-time PCR expression levels. Primers are listed in 5′-3′ orientation.
(DOC)

Table S2 **PAINT analysis of enriched Transcriptional Regulatory Elements (TREs).** PAINT v 3.9, containing a database of promoter sequences (UpstreamDB) constructed for all known and putative annotated genes in the Ensembl genome database for *Homo sapiens*, version 49, cross referenced with Unigene build #213 was used for promoter analysis Statistical significance for TRE overrepresentation was set at p<0.05 with additional filtering performed by setting the false discovery rate (FDR) at 0.3. **N**: enriched in rottlerin treated normal fibroblasts; **S**: enriched in rottlerin treated SSc fibroblasts; **B**: enriched in rottlerin treated normal and SSc fibroblasts.
(DOC)
