# Supplementary material for: Effect of Protein Kinase C delta (PKC-δ) Inhibition on the Transcriptome of Normal and Systemic Sclerosis Human Dermal Fibroblasts In Vitro
Source: PLoS One. 2011 Nov 11;6(11):e27110. doi: 10.1371/journal.pone.0027110 (PMC3214051; doi:10.1371/journal.pone.0027110)
Supplement: Table S1 — Primers used for real-time PCR studies. PCR primers used to validate real-time PCR expression levels. Primers are listed in 5′-3′ orientation. (DOC) [file pone.0027110.s001.doc]

**Supplementary Table 1. Primers used for real-time PCR studies**

| **Gene** | **Forward Primer** | **Reverse Primer** |
| --- | --- | --- |
| **β-actin** | TTGCCGACAGGATGCAGAA | GCCGATCCACACGGAGTACTT |
| **c5orf13** | CACGAACCCAGAGGAGCAG | GGGAGGCAGCGTTTGTCTC |
| **COL1A1** | CCTCAAGGGCTCCAACGAG | TCAATCACTGTCTTGCCCCA |
| **COL3A1** | GGACTGACCAAGATGGGAACAT | GGACTGACCAAGATGGGAACAT |
| **COX1a** | TTCCAATACCGCAACCGCAT | ACTGCTCGTAGCTGTACTCCTG |
| **COX1b** | TTCCAATACCGCAACCGCAT | GGATGTGGTGGTCCATGTTCCT |
| **COX2** | ATCATAAGCGAGGGCCAGCTTTT | AAAGGCGCAGTTTAGGCTGT |
| **CXCL6** | AACCCGCGAACCCTCTCTTG | GCAGCTCTGTCAGCACAGCA |
| **CXCL12** | AAAGCCATGTTGCCAGAGCCAA | AAGTGTGCATTGACCCGAAGCT |
| **DDIT3** | TGGGAGCTGGAAGCCTGGTA | AGCCAAGCCAGAGAAGCAGG |
| **DDIT4** | ACGGTTCGCACACCCATTC | TCAGAGTGCCGGAGCGTAGA |
| **FHL1** | TTGGCCAGATTCACGGAGCATT | TTGTGGCCAAGAAGTGTGCT |
| **FN1** | TTGATGCCGTTCCAGCCAAT | AAACGCAGGTTGGATGGTGCAT |
| **GBP1** | GCCTATGGTGGTGGTGGCAA | ACCGTGGAGCCCAGAGAGAA |
| **GDF15** | TTCCCGGGACCCTCAGAGTT | AGATACGCAGGTGCAGGTGG |
| **IGFBP7** | AGTGCCATGCATCCAATTCCCA | ATAGCTCGGCACCTTCACCTTT |
| **MMP3** | TGGCAGTTTGCTCAGCCTAT | ACAAGGTTGATGCTGGTGTCCT |
| **PLAU** | AGCGACTCCAAAGGCAGCAAT | TTGGGCAGTTGCACCAGTGAAT |
| **PTX3** | TTGGCCGAGACCTCGGATGATT | TGAAGAGCTTGTCCCATTCCGA |
| **RIPK4** | TCAAGCCCGCGAACATCCTG | ATGCGCTCTGGAGGGAGGTA |
| **SOCS2** | TGCAGGGAATGGCAGAGACA | TTACAGGAAGCAGACAGGGCCA |
| **SOX9** | CCAGTACCCGCACTTGCACA | GCCGCGGCTGGTACTTGTAA |
| **THBD** | TACTGGAGCCCAGTCCGTGT | GTTGGCTCTGAAGCACGGGT |
| **TNFRSF19** | TTCCCTGCAACCAGTGTGGG | TCCTTGAACCTGTGCAGCCG |
| **TRIB3** | ACCCAGCTCCTCTACGCCTT | ATCACGCAGGACCAGACCGT |

PCR primers used to validate real-time PCR expression levels. Primers are listed in 5’-3’ orientation.
