# Supplementary material for: Effect of Protein Kinase C delta (PKC-δ) Inhibition on the Transcriptome of Normal and Systemic Sclerosis Human Dermal Fibroblasts In Vitro
Source: PLoS One. 2011 Nov 11;6(11):e27110. doi: 10.1371/journal.pone.0027110 (PMC3214051; doi:10.1371/journal.pone.0027110)
Supplement: Table S2 — PAINT analysis of enriched Transcriptional Regulatory Elements (TREs). PAINT v 3.9, containing a database of promoter sequences (UpstreamDB) constructed for all known and putative annotated genes in the Ensembl genome database for Homo sapiens, version 49, cross referenced with Unigene build #213 was used for promoter analysis Statistical significance for TRE overrepresentation was set at p<0.05 with additional filtering performed by setting the false discovery rate (FDR) at 0.3. N: enriched in rottlerin treated normal fibroblasts; S: enriched in rottlerin treated SSc fibroblasts; B: enriched in rottlerin treated normal and SSc fibroblasts. (DOC) [file pone.0027110.s002.doc]

**Supplementary Table 2 PAINT analysis of enriched Transcriptional Regulatory Elements (TREs)**

| **AHR** | **CCAAT** | **c-ETS1** | **COMP** | **CP2** | **E2F** | **ELK1** | **GR** | **HNF4** | **Myo** | **NFKβ** | **OLF1** | **Pax6** | **STAF** | **TCF11** | **USF** |  |
| --- | --- | --- | --- | --- | --- | --- | --- | --- | --- | --- | --- | --- | --- | --- | --- | --- |
|  |  |  |  |  |  |  |  |  |  |  |  |  |  |  | **S** | **ADORA2** |
|  |  |  |  |  |  |  |  |  |  |  |  |  |  | **N** |  | **AHNAK2** |
|  |  |  | **S** |  |  |  |  | **S** | **S** |  |  |  |  |  |  | **ALDH1L2** |
|  |  |  |  | **S** |  |  |  | **S** |  |  |  |  |  |  |  | **ARG2** |
|  |  |  |  |  | **B** |  |  |  |  |  |  |  |  |  |  | **ARL4C** |
|  | **S** |  | **S** |  |  |  |  |  |  |  |  |  |  |  | **S** | **ASF1B** |
|  |  |  |  |  |  |  |  |  |  |  |  | **S** |  |  |  | **ASN5** |
|  |  |  | **S** |  |  |  |  | **S** |  |  |  |  |  |  |  | **ATP2B1** |
|  |  |  |  |  |  |  |  |  |  |  | **B** |  |  |  |  | **BES2** |
|  |  |  |  |  |  |  |  | **S** |  |  |  |  |  |  |  | **BCAT1** |
|  |  |  |  |  |  |  | **N** |  |  |  |  |  |  |  |  | **BIRC5** |
|  |  |  |  |  |  |  |  |  |  |  |  |  |  |  | **N** | **BNC1** |
|  |  |  |  |  |  |  |  |  |  |  |  |  |  |  | **N** | **BNC2** |
|  | **S** |  |  |  |  |  |  |  |  |  |  |  |  |  | **B** | **C1orf9** |
|  |  |  | **S** |  |  |  |  | **S** |  |  |  |  |  |  |  | **C1orf103** |
|  |  |  |  |  |  |  |  | **S** |  |  |  |  |  |  |  | **C2orf37** |
|  |  |  |  | **N** |  |  |  |  |  |  |  |  |  |  |  | **C3orf55** |
|  |  | **S** | **S** |  |  | **S** |  |  | **S** |  |  |  |  |  |  | **C4orf16** |
|  |  |  | **S** |  |  |  |  |  |  |  |  |  |  |  |  | **C5orf13** |
|  | **S** |  | **S** |  | **N** |  |  | **S** |  |  |  |  |  |  |  | **C6orf105** |
|  |  |  | **S** |  |  |  |  |  |  |  |  |  |  |  |  | **C12orf40** |
|  |  |  |  |  |  |  |  |  |  |  |  |  |  |  | **B** | **CAMK2D** |
|  |  |  |  |  |  |  | **N** |  |  |  |  |  |  |  |  | **CARHSP1** |
|  |  |  |  | **N** |  |  |  |  |  |  |  |  |  |  |  | **CD55** |
|  | **S** |  | **S** |  |  |  |  | **S** |  |  |  |  |  |  |  | **CHAC1** |
|  |  |  | **S** |  |  |  |  |  |  |  |  |  |  |  |  | **CLIC3** |
|  |  |  |  |  |  |  |  | **S** |  |  |  | **S** |  |  | **B** | **COG5** |
|  |  | **S** |  |  |  | **S** |  | **S** |  |  |  |  |  |  |  | **COG6** |
|  |  |  |  |  |  |  |  |  |  |  | **N** |  |  |  |  | **COL13A1** |
|  |  |  | **S** |  |  |  |  |  |  |  |  |  |  |  |  | **COX15** |
|  |  | **S** |  |  |  | **S** |  |  |  |  |  |  |  |  |  | **CPEB4** |
|  |  |  |  |  | **S** | **S** |  |  |  |  |  | **S** |  |  |  | **CSGALNACT2** |
|  |  |  |  | **B** |  |  |  |  |  |  |  |  |  | **N** |  | **CTH** |
|  |  |  | **S** | **S** |  |  |  |  |  |  |  |  |  |  |  | **CXCL6** |
|  |  |  |  |  |  |  |  | **S** |  |  |  |  |  |  |  | **CXCL12** |
|  |  | **S** | **S** | **B** |  | **S** |  |  |  |  |  |  |  |  | **B** | **DDIT3** |
|  |  |  |  |  |  |  |  |  |  |  |  | **S** |  |  |  | **DDIT4** |
|  | **S** |  |  |  |  |  |  | **S** |  |  |  |  |  |  |  | **DFNAS** |
|  |  |  | **S** |  | **S** |  |  |  |  |  |  | **S** |  |  |  | **DIRAS3** |
| **S** |  | **S** |  |  |  |  |  | **S** |  |  |  |  |  |  |  | **DNAJB9** |

| **AHR** | **CCAAT** | **c-ETS1** | **COMP** | **CP2** | **E2F** | **ELK1** | **GR** | **HNF4** | **Myo** | **NFKβ** | **OLF1** | **PAS6** | **STAF** | **TCF11** | **USF** |  |
| --- | --- | --- | --- | --- | --- | --- | --- | --- | --- | --- | --- | --- | --- | --- | --- | --- |
|  |  |  | **S** |  |  | **S** |  | **S** | **S** |  |  |  |  |  |  | **DUSP6** |
|  | **S** | **S** | **S** |  | **S** | **S** |  |  | **S** |  |  |  | **S** |  | **S** | **DYRK3** |
|  | **S** |  | **S** |  |  | **S** |  |  |  |  |  |  |  |  |  | **E1F2AK3** |
|  |  |  |  | **B** |  |  |  |  |  |  |  |  |  |  |  | **E1F4EBP1** |
|  | **S** |  |  |  |  |  |  |  |  |  |  |  |  |  |  | **EAF** |
|  |  |  |  |  | **N** |  |  |  |  |  |  |  |  |  |  | **EAF2** |
|  |  | **S** |  |  |  | **S** |  | **S** |  |  |  |  |  |  |  | **ELOVL6** |
|  |  |  |  |  | **N** |  |  |  |  |  |  |  |  |  |  | **EXO1** |
|  |  |  |  |  |  |  |  | **S** |  |  |  |  |  |  |  | **FAM129A** |
|  |  |  |  |  | **S** | **S** |  |  |  |  | **B** | **S** |  |  | **S** | **FTH1** |
|  |  |  |  |  |  |  |  | **S** |  |  |  |  |  |  |  | **GADD45A** |
|  |  |  |  |  |  |  |  | **S** |  |  |  |  |  |  |  | **GALNT2** |
|  |  |  |  |  | **N** |  |  |  |  |  |  |  |  |  |  | **GARS** |
|  |  |  |  |  |  |  |  | **S** |  |  |  |  |  |  |  | **GAS1** |
|  |  |  |  |  |  |  |  | **S** |  |  |  |  |  |  |  | **GBP1** |
|  |  |  |  |  |  |  |  | **S** |  |  |  |  |  |  |  | **GDF15** |
|  |  |  |  |  |  |  |  | **S** |  |  |  |  |  |  |  | **GFPT1** |
|  |  | **S** |  |  |  | **S** |  |  |  |  |  |  |  |  |  | **GINS2** |
|  | **S** |  | **S** |  | **S** |  |  |  |  |  |  | **S** |  |  |  | **GINS4** |
|  |  |  | **S** |  |  |  |  |  |  |  |  |  |  |  | **S** | **GNA13** |
|  |  |  |  |  |  |  |  |  |  |  |  |  |  |  | **S** | **GNPDA1** |
|  |  |  |  |  |  |  |  | **S** | **S** |  |  | **S** |  |  |  | **GOLSYN** |
|  |  |  |  |  |  |  |  | **S** |  |  |  |  |  |  |  | **GPT2** |
|  | **S** |  |  |  |  |  |  |  |  |  |  |  |  |  |  | **GRP180** |
|  |  | **S** | **S** |  |  | **S** |  |  |  |  |  |  |  |  |  | **GRPEL2** |
|  |  |  |  |  |  |  |  |  |  |  |  |  |  |  | **B** | **HELLS** |
|  |  | **S** | **S** |  |  | **S** |  |  |  |  |  |  |  | **N** |  | **HERPUD1** |
|  |  | **S** |  |  |  | **S** |  | **S** |  |  |  |  |  |  | **S** | **HOMER1** |
|  | **S** |  |  |  |  |  |  | **S** |  |  |  |  |  |  |  | **HSPA5** |
|  |  |  |  | **B** |  |  |  |  |  |  |  |  |  |  |  | **HSPA9** |
|  |  |  |  |  |  |  |  | **S** |  |  |  |  | **S** |  |  | **HSPA13** |
|  |  |  | **S** |  |  |  |  |  |  |  |  |  |  |  |  | **HSPC159** |
| **S** |  |  |  |  |  |  |  |  |  |  |  |  |  |  |  | **HUNK** |
|  |  |  |  |  |  |  |  | **S** |  |  |  |  |  |  |  | **IGDCC4** |
|  | **S** |  |  |  |  |  |  |  |  |  |  |  |  |  |  | **IL8** |
|  |  |  |  |  |  |  |  |  |  |  |  | **S** | **S** |  |  | **JARID2** |
|  |  |  |  |  |  |  |  |  |  |  |  |  |  |  | **B** | **KCNG1** |
|  |  |  |  | **B** |  |  |  |  |  |  |  |  |  |  |  | **KDSR** |
|  |  |  | **S** |  |  |  |  |  |  |  |  |  |  |  |  | **KLH** |
|  |  |  | **S** |  |  |  |  |  |  |  |  |  |  |  |  | **LRP8** |

| **AHR** | **CCAAT** | **c-ETS1** | **COMP** | **CP2** | **E2F** | **ELK1** | **GR** | **HNF4** | **Myo** | **NFKβ** | **OLF1** | **PaS6** | **STAF** | **TCF11** | **USF** |  |
| --- | --- | --- | --- | --- | --- | --- | --- | --- | --- | --- | --- | --- | --- | --- | --- | --- |
|  |  |  |  |  |  |  |  | **S** |  |  |  |  | **S** |  |  | **MARS** |
|  |  |  |  |  | **S** |  |  |  |  |  |  |  |  |  |  | **MCM4** |
|  | **S** |  | **S** |  | **B** |  |  |  |  |  |  | **S** |  |  |  | **MCM5** |
|  |  |  |  |  |  |  |  |  |  |  |  |  |  |  | **N** | **METRNL** |
|  |  |  |  |  |  |  |  |  |  |  |  |  |  |  | **N** | **MFHAS1** |
|  |  |  |  |  |  |  |  | **S** |  |  |  |  |  |  |  | **MKNK2** |
|  |  |  | **S** |  |  |  |  |  |  |  |  |  |  |  |  | **MSC** |
|  |  |  |  | **B** |  |  |  |  |  |  |  |  |  |  |  | **MTHFD2** |
|  |  |  | **S** |  |  |  |  | **S** |  |  |  |  |  |  |  | **MT1F** |
|  | **S** |  | **S** |  |  | **S** |  |  |  | **B** |  |  |  |  |  | **MT1X** |
|  |  |  | **S** |  |  |  |  |  |  |  | **B** |  |  |  |  | **MTSS1** |
|  |  |  | **S** |  |  |  |  |  |  |  |  |  |  |  |  | **MXD1** |
|  |  |  | **S** |  |  |  |  | **S** |  |  |  |  |  |  |  | **NAMPT** |
|  |  |  |  | **N** |  |  |  |  |  |  |  |  |  |  |  | **NCAPG** |
|  |  |  |  |  |  |  |  |  |  |  |  | **S** |  |  |  | **NHLRC3** |
|  |  | **S** |  |  |  | **S** |  | **S** |  |  |  |  |  |  |  | **NPC1** |
|  |  |  | **S** |  |  |  |  |  |  |  |  |  |  |  | **B** | **NRBF2** |
|  |  |  |  |  |  |  |  | **S** |  |  |  | **S** |  |  |  | **NRP2** |
|  |  |  |  |  |  |  |  | **S** |  |  |  |  |  |  |  | **NUPR1** |
|  |  |  |  |  | **N** |  |  |  |  |  |  |  |  |  |  | **NUSAP1** |
|  |  |  |  |  |  |  |  |  |  |  |  |  |  |  | **N** | **OSBPL8** |
|  |  |  |  |  |  | **S** |  |  |  |  |  |  |  |  |  | **PAPOLA** |
|  | **S** |  | **S** |  |  |  |  |  |  |  |  |  |  |  |  | **PCDH18** |
|  |  |  |  |  |  |  |  | **S** |  |  |  | **S** |  |  |  | **PCK2** |
|  |  |  |  | **B** |  |  |  |  |  |  |  |  |  |  |  | **PHGDH** |
|  |  |  |  |  |  |  |  | **S** |  |  |  |  |  |  |  | **PHLDA1** |
|  |  |  |  |  |  |  |  |  | **S** |  |  | **S** |  |  |  | **PLAUR** |
|  |  |  |  |  |  |  |  |  |  |  |  | **S** |  |  |  | **PLECKHF1** |
|  |  |  |  |  |  |  |  | **S** |  |  |  |  |  |  |  | **PPM1K** |
|  |  |  |  |  |  |  |  | **S** | **S** |  |  |  |  |  |  | **PTER** |
|  |  |  |  |  |  |  |  | **S** |  |  |  |  |  |  |  | **PTGS2** |
|  |  |  | **S** |  |  |  |  |  |  |  |  |  | **S** |  |  | **RAB7L1** |
|  |  |  |  |  |  |  |  | **S** |  |  |  | **S** |  |  |  | **RAB33A** |
|  |  |  |  |  |  |  |  | **S** |  |  |  |  |  | **N** | **B** | **RCAN1** |
|  |  | **S** |  | **S** |  | **S** |  | **S** |  | **S** |  | **S** |  |  | **S** | **RCHY1** |
|  |  |  |  |  |  |  |  | **S** |  |  |  |  |  |  |  | **RIPK4** |
|  |  |  | **S** |  |  | **S** |  |  |  |  |  |  |  |  | **S** | **RMND1** |
|  |  |  |  |  |  |  |  |  |  |  |  | **S** |  |  |  | **ROGDI** |
|  | **S** |  |  |  |  |  |  | **S** |  |  |  |  | **S** |  |  | **SESN2** |
|  |  |  |  |  |  |  |  |  |  |  |  |  |  |  | **N** | **SH2D2** |

| **AHR** | **CCAAT** | **c-ETS1** | **COMP** | **CP2** | **E2F** | **ELK1** | **GR** | **HNF4** | **Myo** | **NFKβ** | **OLF1** | **PaS6** | **STAF** | **TCF11** | **USF** |  |
| --- | --- | --- | --- | --- | --- | --- | --- | --- | --- | --- | --- | --- | --- | --- | --- | --- |
|  |  |  |  |  | **N** |  |  |  |  |  |  |  |  |  |  | **SHMT2** |
|  |  |  |  |  |  |  |  |  | **S** | **B** |  |  |  |  |  | **SHROOM3** |
|  |  |  | **S** |  |  |  |  |  |  |  |  |  |  |  |  | **SLC1A4** |
|  |  |  |  |  |  |  |  |  | **S** |  |  |  |  |  |  | **SLC2A13** |
|  | **S** |  |  |  | **N** |  |  |  |  |  |  | **S** |  |  | **B** | **SLC3A2** |
|  |  |  |  |  |  |  |  |  | **S** |  |  |  |  |  | **S** | **SLC6A15** |
|  |  |  |  |  |  |  |  | **S** |  |  |  |  |  |  |  | **SLC7A5** |
|  |  |  | **S** |  |  |  |  | **S** |  |  |  |  |  |  |  | **SLC7A11** |
|  |  |  |  |  |  |  |  |  |  |  |  |  |  | **N** |  | **SLC25A36** |
|  |  | **S** |  |  |  | **S** |  |  |  |  |  |  |  |  |  | **SLC31A1** |
|  |  |  |  | **S** |  |  |  | **S** |  |  |  |  | **S** |  |  | **SLC38A1** |
|  |  |  |  |  |  | **S** |  |  |  |  |  |  |  |  |  | **SLFN5** |
|  |  |  |  |  |  |  | **N** |  |  |  |  |  |  |  |  | **SLMO2** |
|  |  |  |  |  |  |  |  | **S** |  |  |  |  |  |  |  | **SMYD3** |
|  |  |  | **S** |  |  |  |  | **S** |  |  |  |  | **S** |  |  | **SQSTM1** |
|  |  |  |  |  |  |  |  | **S** |  |  |  |  |  |  |  | **STX3** |
|  |  |  |  | **N** | **N** |  |  |  |  | **B** |  |  |  |  | **N** | **SSBP3** |
|  |  |  |  |  |  |  |  |  |  |  |  |  |  |  | **N** | **TACC3** |
|  |  |  |  | **N** |  |  |  |  |  |  |  |  |  |  |  | **TCF4** |
|  |  |  |  | **N** |  |  |  |  |  |  |  |  |  |  |  | **TCF19** |
|  |  |  |  |  | **S** |  |  |  |  |  |  | **S** |  |  |  | **TEAD2** |
|  |  |  |  |  |  | **S** |  |  |  |  |  |  |  |  |  | **TGIF1** |
|  |  |  |  |  |  |  |  |  |  |  |  | **S** |  |  |  | **TGDS** |
|  |  |  | **S** |  | **S** |  |  |  |  |  |  | **S** |  |  |  | **TMEM38B** |
|  | **S** |  |  |  |  |  |  |  |  |  |  |  |  |  |  | **TNFRSF19** |
|  |  |  |  |  |  |  |  |  |  |  |  | **S** |  |  |  | **TRIB3** |
|  |  |  |  |  |  |  |  |  |  |  |  |  |  |  | **N** | **TRIM25** |
|  |  |  | **S** |  |  |  |  | **S** |  |  |  |  |  |  |  | **TRPA1** |
|  |  |  |  | **B** |  |  |  | **S** |  |  |  |  |  |  | **B** | **UAP1L1** |
|  |  |  | **S** |  | **S** |  |  | **S** |  |  |  |  |  |  |  | **UHRF1** |
|  |  |  |  |  |  |  |  |  |  |  |  | **S** |  |  |  | **UPS37A** |
|  |  |  |  |  |  |  |  |  |  |  |  | **S** |  |  |  | **VEGFA** |
|  | **S** | **S** |  |  |  | **S** |  |  |  |  |  |  |  |  |  | **WARS** |
|  |  |  |  |  | **N** |  |  |  |  |  |  |  |  |  | **N** | **WBP2** |
|  |  |  |  |  |  | **S** |  | **S** |  |  |  |  |  |  |  | **WIPI1** |
|  |  |  |  |  |  |  |  |  |  |  |  |  |  |  | **N** | **WISP1** |
|  |  |  |  |  |  | **S** |  |  |  |  |  |  |  |  |  | **XPOT** |
|  |  |  |  |  |  | **S** |  |  |  |  |  |  |  |  |  | **ZNF295** |

**N**: enriched in rottlerin treated normal fibroblasts; **S**: enriched in rottlerin treated SSc fibroblasts; **B**: enriched in rottlerin treated normal and SSc fibroblasts.
